# Parallel-Disk Viscometry of a Viscoplastic Hydrogel: Yield Stress and Other Parameters of Shear Viscosity and Wall Slip †

**DOI:** 10.3390/gels8040230

**Published:** 2022-04-07

**Authors:** Li Quan, Dilhan M. Kalyon

**Affiliations:** 1 Department of Chemical Engineering and Materials Science, Stevens Institute of Technology, Hoboken, NJ 07030, USA; lquan1@stevens.edu; 2 Highly Filled Materials Institute, Stevens Institute of Technology, Hoboken, NJ 07030, USA

**Keywords:** hydrogel, gel, microgel, wall slip, viscoplastic, plug flow, continuous deformation, parallel disk, viscometry

## Abstract

The rheology, i.e., the flow and deformation properties, of hydrogels is generally a very important consideration for their functionality. However, the accurate characterization of their rheological material functions is handicapped by their ubiquitous viscoplasticity and associated wall slip behavior. Here a parallel-disk viscometer was used to characterize the shear viscosity and wall slip behavior of a crosslinked poly(acrylic acid) (PAA) carbomer hydrogel (specifically Carbopol^®^ at 0.12% by weight in water). It was demonstrated that parallel-disk viscometry, i.e., the steady torsional flow in between two parallel disks, can be used to unambiguously determine the yield stress and other parameters of viscoplastic constitutive equations and wall slip behavior. It was specifically shown that torque versus rotational speed information, obtained from parallel-disk viscometry, was sufficient to determine the yield stress of a viscoplastic hydrogel. Additional gap-dependent data from parallel-disk viscometry could then be used to characterize the other parameters of the shear viscosity and wall slip behavior of the hydrogel. To investigate the accuracy of the parameters of shear viscosity and apparent wall slip that were determined, the data were used to calculate the torque values and the velocity distributions (using the lubrication assumption and parallel plate analogy) under different flow conditions. The calculated torques and velocity distributions of the hydrogel agreed very well with experimental data collected by Medina-Bañuelos et al., 2021, suggesting that the methodologies demonstrated here provide the means necessary to understand in detail the steady flow and deformation behavior of hydrogels. Such a detailed understanding of the viscoplastic nature and wall slip behavior of hydrogels can then be used to design and develop novel hydrogels with a wider range of applications in the medical and other industrial areas, and for finding optimum conditions for their processing and manufacturing.

## 1. Introduction

### 1.1. Gels and Gelation

Both physical and chemical gelation processes are used to generate gels, which exhibit flow and deformation behavior resembling solid elastic bodies and viscous fluids under differing flow conditions. Chemical gelation typically involves a polymerization process whereby the macromolecules are connected (crosslinked) via covalent bonds [1]. Up to a certain degree of conversion the macromolecules are soluble (sol phase) whereas with increasing conversion the macromolecules form a three-dimensional network that spans the entire volume of the sample (gel phase) [1]. As the crosslink density increases during chemical gelation, crosslinked polymer clusters are formed and the cluster size increases with increasing degree of crosslinking. When only parts of the polymer molecules crosslink and span the volume, with sol phases in between the macromolecules, a “microgel” is formed [2].

On the other hand, gelation also occurs via physical mechanisms whereby, for example, clusters of particles consisting of crosslinked polymers, start to interact with each other via dipole-dipole interactions, traces of crystallinity, van der Waals interactions [3,4,5,6,7], surface chemistry [8,9,10], hydrogen bonding-based complexation [11], hydrophobic effects [12] and depletion interactions [13,14,15]. Important attraction mechanisms that drive gelation are generally short range, and include “van der Waals forces, surface chemistry, hydrophobic effects, and some depletion interactions” [4]. During the gelation process when macromolecules go from the sol phase to the gel phase, with various states of microgel formation, the linear viscoelastic properties are good reflections of the structural changes arising from the sol to gel transition [11,16,17,18,19]. Fully formed gels can be highly elastic.

**Figure 1:**
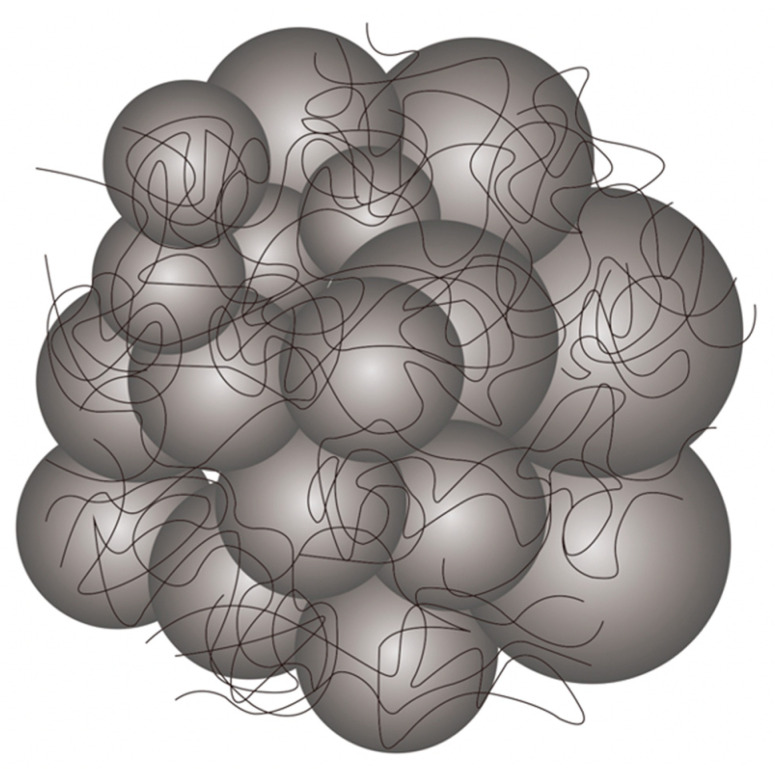
A schematic representation of the structure of close-packed crosslinked and swollen Carbopol^®^ gel particles. (Adapted from Shafiei et al. [20] with permission from Elsevier).

An interesting gelation agent that is widely used for various industrial applications is carbomer (for example, Carbopol^®^, which is a tradename of the Lubrizol Corporation of Wickliffe, Ohio USA, that is used in our study) which consists of poly(acrylic acid) (PAA) molecules crosslinked into spherical clusters, i.e., soft particles (Figure 1). Typically, Carbopol^®^ particles are swollen in water, with a water-rich continuous phase in between the swollen particles [20,21].

**Figure 2:**
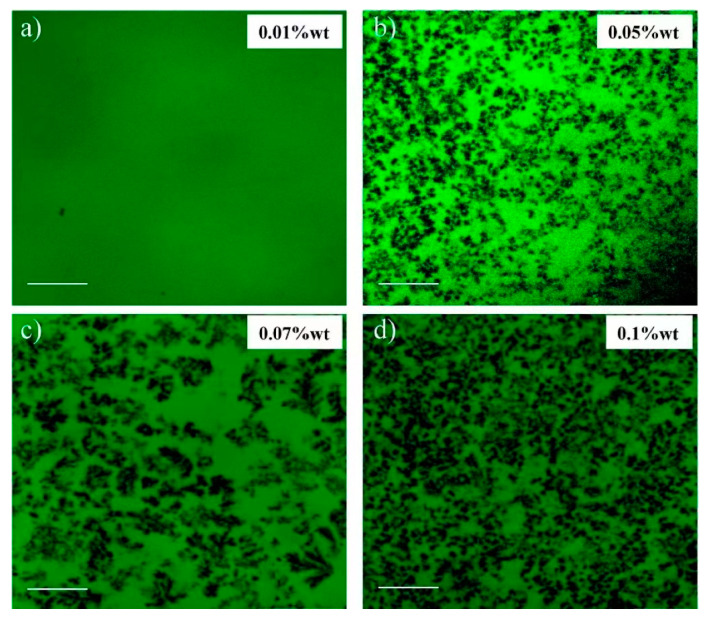
Fluorescence micrographs of Carbopol^®^ hydrogels at various concentrations of Carbopol^®^ particles in water phase indicating particle cluster formation and networking with increasing concentration (**a**) 0.01%, (**b**) 0.05%, (**c**) 0.07% and (**d**) 0.1 wt% [22]. The scale bar corresponds to 10 μm. Reproduced from Graziano et al. [22] with permission from Elsevier.

Aqueous dispersions of such crosslinked polymer gels can be prepared over a range of conditions, concentrations, and pH. Figure 2 shows the fluorescence micrographs of Carbopol^®^ hydrogels at various concentrations of Carbopol^®^ [22]. At low concentrations of Carbopol^®^ there are no visible interactions and clustering of swollen particles. However, when the concentration of the Carbopol^®^ reaches 0.1% by weight agglomeration and clustering of the soft particles can be observed [22]. In fact, at 0.1% by weight (Figure 2d), the particle clusters span the length of the sample to generate a microgel. The onset of the jamming of the swollen particles with increasing concentration gives rise to elasticity and gel-like behavior. Such network formation is the basis for the development of a yield stress for the hydrogel which demarcates the boundary between solid-like and fluid-like behavior. Piau observed that the clustering of the crosslinked particles can span the entire volume above a critical concentration at which a percolated network is developed [23].

### 1.2. Viscoplasticity and Wall Slip of Hydrogels

Carbomer hydrogels (microgels and gels of swollen PAA particles in water) are used in many applications including as thickeners in personal care products [24,25,26]. The rheological behavior of Carbopol^®^ hydrogels at concentrations around 0.1% and higher have been investigated extensively due to their viscoplastic nature with their flow and deformation behavior affected by the stress field that is acting on the hydrogel during flow [23]. For example, in steady simple shear flow (only one component of the velocity vector prevails and depends only on one other direction), when the absolute value of the shear stress that is applied continuously during simple shear flow is smaller than the yield stress of the hydrogel, the hydrogel does not deform continuously. Under such conditions, plug flow, enabled by slip at the wall, is observed [27,28]. When the shear stress is greater than the yield stress of the hydrogel there is continuous deformation of the gel (the hydrogel deforms at a constant deformation rate as long as the shear stress is applied) accompanied by slip at the wall [28,29].

Thus, the wall slip and deformation behaviors of viscoplastic fluids, including viscoplastic hydrogels, are coupled and need to be investigated concomitantly [27,29,30,31,32,33,34,35,36,37,38,39,40]. Generally, the wall slip of complex fluids, including suspensions with soft or hard particles and gels, occurs via an apparent slip mechanism [27,30,41,42,43] which can also be affected by the presence of a gas phase, for example, air entrainment [44,45,46,47,48]. Such apparent slip layer formation can also be influenced by the migration of particles away from high shear rate regions [49,50,51,52,53,54]. The use of roughened surfaces to eliminate wall slip can lead to the fracture of the viscoplastic fluid [34,55].

There are significant ramifications of apparent wall slip and viscoplastic behavior in complex flows and in the processing of various complex fluids [29,30,36,37,38,56,57,58,59,60,61,62,63,64,65,66,67,68,69,70,71,72,73,74,75]. The wall slip of the polymer phase itself is typically observed above a critical shear stress. Such slip at the wall of the polymeric binder gives rise to processing difficulties and challenges, including development of flow instabilities that change the nature of the shape of the extrudates emerging from pressure-driven flows, such as shark skin and gross melt fracture, and time-dependence of the pressure applied to drive the flow [67,73,76,77,78,79,80,81].

The flow and deformation behavior of Carbopol^®^ hydrogels have been investigated in detail previously via flow-through capillary and rectangular-slit dies [29,82], axial annular flow (flow in between two stationary cylinders as a result of a pressure gradient) [83], Couette flow (double coaxial cylinders, one of which is rotating and the other is stationary) [3,84,85,86,87,88,89,90,91], and vane-in-cup flow [92,93,94,95,96,97]. These viscometric flows have demonstrated the viscoplastic nature of the Carbopol^®^ hydrogels whereby the yield stress value of the hydrogel could be determined unambiguously in conjunction with the wall slip behavior of the hydrogel. In the following, an in-depth analysis of parallel-disk viscometry (steady torsional flow) is carried out to demonstrate how the yield stress of the hydrogel can be determined using parallel-disk viscometry, followed by characterization of the other parameters of shear viscosity and wall slip, prediction of the torque and velocity distributions in between the two parallel disks, and comparison of the predictions of velocity distributions and torques with the experimental values that were available from Medina-Bañuelos et al., 2022 [98].

### 1.3. Parallel-Disk Viscometry (Steady Torsional Flow)

**Figure 3:**
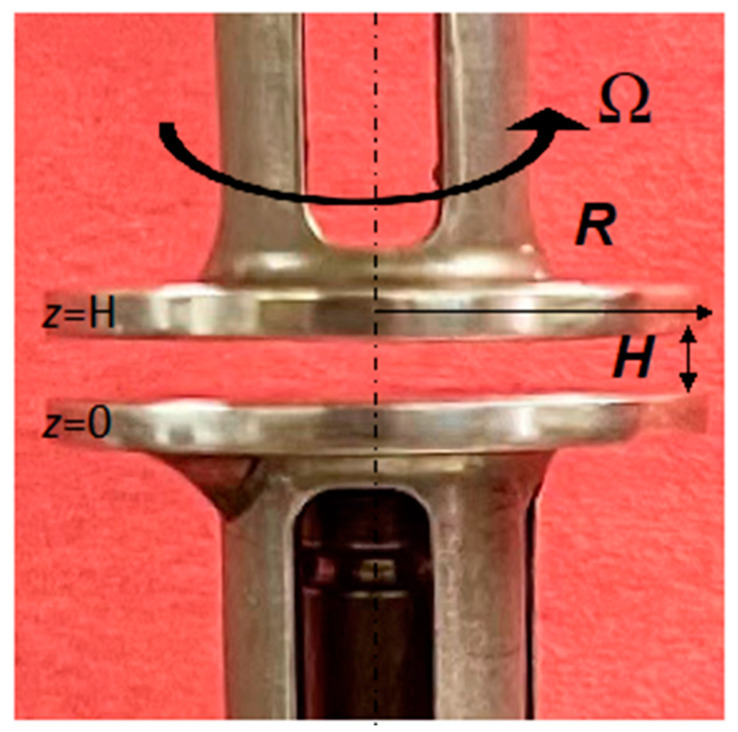
Steady torsional flow between two parallel disks with gap, *H*, radius, *R* and Ω is the rotational speed.

Parallel disk viscometry is one of the simplest geometries that can be used for the rheological characterization of complex fluids (steady torsional flow in between two parallel disks) (Figure 3) in which the sample is sandwiched in between two disks, one of which is rotating at a rotational speed of Ω, and the other is stationary. The gap, *H*, in between the two disks is typically significantly smaller than the radius of the disks, *R*, i.e., *H << R*. The condition, *H << R*, results in the shear stress component associated with the velocity gradient in the depth direction to be significantly greater than the shear stress component that exists in the radial direction, so that the flow can be considered to be a simple shear flow (one component of the velocity vector, *V_θ_*, changing in only one other direction, *z*) and a simple parallel plate analysis of the torsional flow can be carried out employing the lubrication assumption [99]. It is also possible to use a cone-and-plate fixture whereby the sample is sandwiched in between a cone with a cone angle of *α* and a disk. The cone-and-plate geometry is very suitable for the characterization of the rheological behavior of Newtonian and generalized Newtonian fluids that do not exhibit viscoplasticity nor wall slip [100], since the shear rate and the shear stress are constant within the gap for simple fluids [101,102]. However, for various complex fluids which exhibit wall slip, such as viscoplastic hydrogels, the flow curves are dependent on the radial location and thus the cone-and-plate geometry offers no advantages [103].

In the following, first the torque versus the apparent shear rate behavior in steady torsional flow of a Carbopol^®^ hydrogel (at 0.12% by weight Carbopol^®^ in water), that was investigated earlier for its Couette [84], and vane-in-cup [92] flows, are analyzed to generate the parameters of a viscoplastic constitutive equation starting with the yield stress (Herschel–Bulkley) and the parameters describing the wall slip velocity versus shear stress relationship. It is shown here that the torque versus apparent shear rate data collected with the parallel-disk viscometry allow, in a relatively facile manner, the determination of the yield stress of a viscoplastic hydrogel, consistent with similar findings from the steady torsional flow of suspensions containing rigid particles [99]. It will be demonstrated that the other parameters of the Herschel–Bulkley fluid constitutive equation can then be determined from the flow curves employing wall slip analysis.

### 1.4. Yield Stress

As noted earlier, when subjected to simple shear flows, viscoplastic fluids exhibit solid-like behavior (plug flow) when the shear stress that is applied is less than a critical value, i.e., the yield stress $\tau_{0}$, and a constant deformation rate when the shear stress applied is above the yield stress. The determination of the yield stress is a challenge and is one of the most misunderstood concepts in the field of rheology [32]. The shear stress growth and the shear stress relaxation upon cessation of steady shear are suggested to be used for the determination of the yield stress values of viscoplastic materials [104]. For example, the residual “limiting” shear stress exerted by the fluid upon the cessation of the steady shear flow is suggested to represent the yield stress [105]. This assumes that the value of the limiting stress would be insensitive to the constant deformation rate. However, as noted by Magnin and Piau (1990) [106], in the presence of wall slip, the limiting stress becomes a function of the imposed constant deformation rate which complicates the determination of the yield stress value. Another method is to use the extrapolation of the shear stress versus the apparent shear rate data to diminishing shear rates to identify the yield stress. However, the yield stress values determined as such become dependent on the surface-to-volume ratio of the rheometer geometry used [32]. Finally, roughened rheometer surfaces are suggested to be used to obtain the flow curve directly (shear stress versus the shear rate), with the stipulation that rough surfaces will eliminate wall slip so that the apparent shear rate becomes equal to the true shear rate. The fitting of the flow curve thus obtained would provide the yield stress. However, it has been shown that the roughening of the rheometer surfaces can lead to the fracturing of the viscoplastic fluid, rendering the data meaningless [34,55].

In the following, steady torsional flow (parallel-disk viscometry) is used to determine the yield stress and other viscoplastic flow (Herschel–Bulkley) and wall slip parameters of a Carbopol^®^ hydrogel. The parameters of shear viscosity and wall slip of the hydrogel were used and tested via predictions of the velocity distributions and torques that were then compared with the recently published experimental velocity distributions and torque results of Medina-Bañuelos et al. [98]. The methods presented here for the characterization of the flow and deformation behavior of viscoplastic fluids have also been tested earlier for a concentrated suspension of rigid particles which also exhibited viscoplasticity and apparent wall slip [99]. Overall, the proposed procedure should significantly simplify the characterization of the yield stress values of viscoplastic hydrogels. The analysis results also provide a better understanding of the flow and deformation behavior of viscoplastic hydrogels in general, and their steady torsional flow in particular, for example, by allowing the determination of the shear stress distributions as a function of the radial distance, *r*, and the resulting velocity distributions and torques under various flow conditions in steady torsional flow.

### 1.5. Background

#### 1.5.1. Viscoplastic Constitutive Equation (Herschel–Bulkley)

Let us start via the formal definition of the flow and deformation behavior of viscoplastic fluids. Viscoplasticity mandates that under steady-state conditions the flow behavior of viscoplastic fluids is binary in nature, i.e., that the deformation rate as represented by the rate of deformation tensor, $\underline{\Delta }$, is zero when the stress magnitude is less than the yield stress, i.e., $1/2(\underline{\tau }:\underline{\tau })<\tau_{0}^{2}$:

(1) $$\underline{\Delta }=0\;\mathrm{for}\;1/2(\underline{\tau }:\underline{\tau })\leq \tau_{0}^{2}$$

and is finite when the stress magnitude is greater than the yield stress, i.e., for the condition $1/2(\underline{\tau }:\underline{\tau })>\tau_{0}^{2}$:

(2) $$\underline{\tau }=-\eta \left(II_{\Delta }\right)\underline{\Delta }\;\mathrm{for}\;1/2(\underline{\tau }:\underline{\tau })>\tau_{0}^{2}$$

where the shear viscosity, *η*, is a function of the second invariant of the rate of deformation tensor, $II_{\Delta }$, i.e., $\eta \left(II_{\Delta }\right)$. Equation (2) is the generalized Newtonian fluid model, which stipulates that the stress tensor is equal to the rate of deformation tensor times the shear viscosity material function under steady flow conditions [101,102].

It was shown for various viscometric and processing flows that the Herschel–Bulkley Equation accurately represents the behavior of various viscoplastic fluids for $1/2(\underline{\tau }:\underline{\tau })>\tau_{0}^{2}$ [6,29,36,38,81,83,84,92,101,102], i.e.,

(3) $$\underline{\tau }=-\left(\frac{\tau_{0}}{\left|\sqrt{1/2(\underline{\Delta }:\underline{\Delta })}\right|}+m{\left|\sqrt{1/2(\underline{\Delta }:\underline{\Delta )}}\right|}^{n-1}\right)\underline{\Delta }\;\mathrm{for}\;1/2(\underline{\tau }:\underline{\tau })>\tau_{0}^{2}$$

The Herschel–Bulkley Equation involves three parameters at constant temperature, i.e., the yield stress, $\tau_{0}$, the consistency index, *m*, and the shear rate sensitivity index, *n* (also referred to as the power law index, indicating Newtonian behavior above the yield stress, i.e., Bingham fluid, for *n* = 1, or shear thinning or shear thickening with *n* < 1 or *n* > 1, respectively). For steady torsional flow the Herschel–Bulkley Equation becomes:

(4) $$\tau_{z\theta }\left(r\right)=\pm \tau_{0}-m{\left|\frac{dV_{\theta }}{dz}\left(r\right)\right|}^{n-1}\left(\frac{dV_{\theta }}{dz}\left(r\right)\right)\;\mathrm{for}\;\left|\tau_{z\theta }\left(r\right)\right|>\tau_{0}$$

(5) $$\frac{dV_{\theta }}{dz}\left(r\right)=0\;\mathrm{for}\;\left|\tau_{z\theta }\left(r\right)\right|\leq \tau_{0}$$

where $\frac{dV_{\theta }}{dz}\left(r\right)$ and $\tau_{z\theta }\left(r\right)$ are the true shear rate and the shear stress for any radial position, *r*, respectively [27,29]. In Equation (4) the − sign is used when the shear stress, $\tau_{z\theta }\left(r\right)$ is negative. Considering the case of the top disk rotating, so that $\tau_{z\theta }\left(r\right)<0$, Equation (4) becomes: $\tau_{z\theta }\left(r\right)=-\tau_{0}-m{\left|\frac{dV_{\theta }}{dz}\left(r\right)\right|}^{n-1}\left(\frac{dV_{\theta }}{dz}\left(r\right)\right)=-\tau_{0}-m{\left(\frac{dV_{\theta }}{dz}\left(r\right)\right)}^{n}$.

#### 1.5.2. Wall Slip Velocities in Steady Torsional Flow (Parallel-Disk Viscometry)

**Figure 4:**
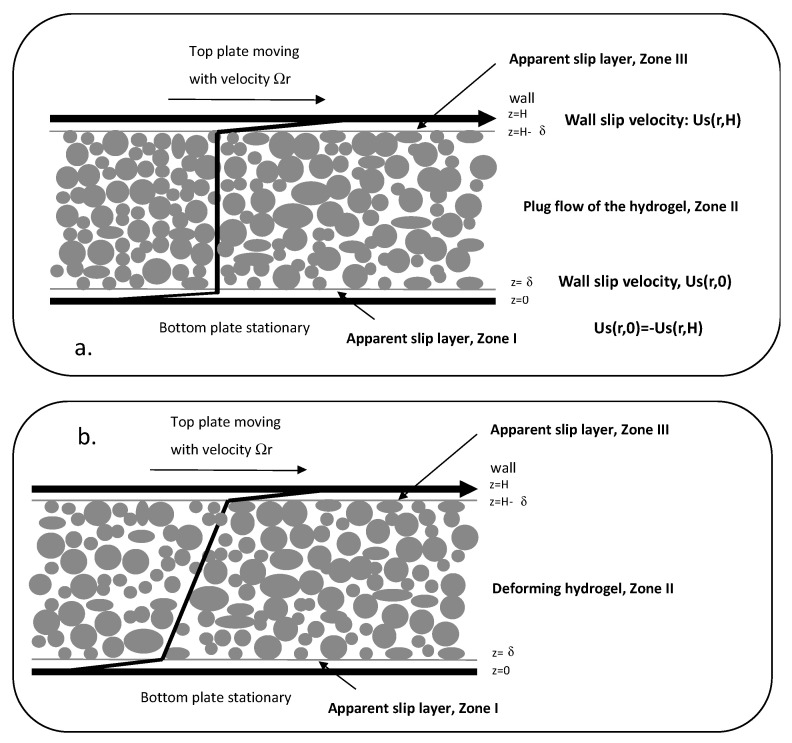
Schematics of the steady torsional flow of a viscoplastic hydrogel (**a**) plug flow with apparent slip (**b**) continuous deformation with apparent slip at the walls.

Figure 4a,b show the schematics of the velocity distributions for a viscoplastic fluid during steady torsional flow as depicted via the parallel-plate flow assumption. Under the conditions of the shear stress, $\left|\tau_{z\theta }\left(r\right)\right|\leq \tau_{0}$, plug flow occurs (Figure 4a) and for $\left|\tau_{z\theta }\left(r\right)\right|>\tau_{0}$, a constant deformation rate prevails for the viscoplastic fluid in between the two walls as long as a constant shear stress is imposed (Figure 4b).

The apparent wall slip of viscoplastic fluids is also schematically shown in Figure 4, where apparent slip layers are depicted in an exaggerated manner at both the top and bottom surfaces [27]. The wall slip velocity $U_{s}$ is defined as the difference between the velocity of the fluid at the wall, and the velocity of the wall. Thus, the wall slip velocity is negative for the top disk which is moving, i.e., $U_{s}\left(r,H\right)<0$, and the wall slip velocity, $U_{s}\left(r,0\right)$, is positive for the bottom disk, which is stationary, i.e., $U_{s}\left(r,0\right)>0$. The wall slip velocities at the top and bottom disks are related to each other as $U_{s}\left(r,H\right)=-U_{s}\left(r,0\right)$ For similar wall slip behavior at the top and bottom surfaces, the slip velocity for plug flow conditions $\left|\tau_{z\theta }\left(r\right)\right|\leq \tau_{0}$ is equal to the wall velocity, $V_{w}=\Omega R$, over two, i.e., $U_{s}=\Omega R/2$ [27]. Thus, for plug flow, the slip velocities at the bottom and top surfaces are only a function of the plate velocity, Ωr [27],

(6) $$U_{s}\left(r,0\right)=\frac{\Omega r}{2}\;\mathrm{and}\;U_{s}\left(r,H\right)=-\frac{\Omega r}{2}$$

For the rest of the manuscript, “wall slip velocity” will refer to the absolute values of the wall slip velocities at the top and bottom surfaces to avoid confusion.

#### 1.5.3. Apparent Slip Flow Mechanism

The wall slip behavior of various viscoplastic fluids, including concentrated suspensions and gels with rigid and soft particles, is subject to the apparent slip mechanism. During the flow of a suspension or gel of rigid or soft particles the particles cannot physically occupy the space adjacent to a wall as efficiently as they can away from the wall [27,41,42,43]. This leads to the formation of a, generally relatively thin, but always present, layer of pure fluid adjacent to the wall, i.e., the “apparent slip layer” or the “Vand layer” [107]. The lower viscosity at the particle-free apparent slip layer gives rise to a higher shear rate at the wall at a given shear stress and hence gives the appearance of wall slip, considering that the slip layer thickness is much smaller than the channel dimension, i.e., apparent wall slip [27,30,41,42].

For suspensions of rigid particles, the estimates of the slip layer thickness over the particle diameter ratio are available [27,30,85,90,91,108]. Meeker et al. have shown that the apparent slip mechanism is also applicable to microgel pastes and concentrated emulsions and have provided methods for the estimation of the apparent slip layer thickness, *δ*, based on elastohydrodynamic lubrication between squeezed soft particles and shearing surfaces [90,91]. For viscoplastic microgels the apparent slip mechanism could be integrated into the analysis of various flows including steady torsional, capillary, tangential annular (Couette), axial annular and vane-in-cup flows [29,83,84,92].

The relationship between the slip velocity, $U_{s}\left(\tau_{z\theta }\left(r\right)\right)$, and the shear stress, $\tau_{z\theta }\left(r\right)$, for apparent wall slip occurring in steady torsional flow becomes the following (top surface rotating) for Vand layers, the shear viscosity of which can be described by a power law equation represented with a consistency index, *m_b_*, and a power law index of *n_b_*, i.e., $\tau_{z\theta }\left(r\right)=-m_{b}{\left|\frac{dV_{\theta }}{dz}\left(r\right)\right|}^{n_{b}-1}\left(\frac{dV_{\theta }}{dz}\left(r\right)\right)=-m_{b}{\left(\frac{dV_{\theta }}{dz}\left(r\right)\right)}^{n_{b}}$, as [27]:

(7) $$U_{s}\left(r,0\right)=\frac{\delta }{m_{b}{}^{1/n_{b}}}{\left(-\tau_{z\theta }\left(r\right)\right)}^{1/n_{b}}\;\mathrm{and}\;U_{s}\left(r,H\right)=-\frac{\delta }{m_{b}{}^{1/n_{b}}}{\left(-\tau_{z\theta }\left(r\right)\right)}^{1/n_{b}}$$

For suspensions of rigid, low-aspect-ratio and non-colloidal particles in the volume fraction of solids, range of 0.17 to 0.94, compilation of apparent slip layer thickness data over a wide range of concentrations has indicated that the apparent slip layer can be related to the harmonic mean particle diameter and the ratio of the volume fraction of solids over their maximum packing fraction, i.e., $\frac{\phi }{\phi_{m}}$, and can be determined from: $\frac{\delta }{D_{p}}=\left(1-\frac{\phi }{\phi_{m}}\right)$ [27,109,110]. For pressure-driven flows the apparent wall slip behavior under the plug flow conditions can be complicated and the apparent slip layer can be a function of the flow rate [56]. Such dependence on the flow conditions can be a consequence of the binder itself exhibiting wall slip, which typically occurs at shear stresses that are above a critical wall shear stress [67,81,111,112,113]. However, such complications are not observed for Newtonian binders [27,30,40,85]. It should be noted that, in the following, additional light will be shed onto the nature of the apparent wall slip mechanism for carbomer hydrogels.

## 2. Results and Discussion

### 2.1. Parallel-Disk Viscometry Yield Stress from Torque versus Apparent Shear Rate

**Figure 5:**
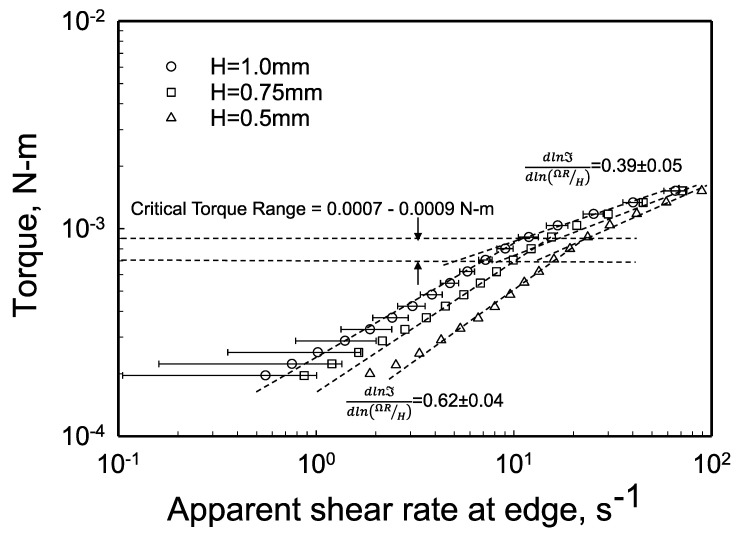
Steady torque, ℑ, versus the apparent shear rate at the edge for three gaps. The critical torque corresponds to the yield condition from which the yield stress can be determined.

The experimental torque, ℑ, versus the apparent shear rate data (${\dot{\gamma }}_{aR}=\Omega R/H$) from parallel-disk viscometry are shown in Figure 5 for three gaps of 0.5, 0.75 and 1 mm [98]. The data were best fitted to determine the variation of the slope, $\frac{d\ln ℑ}{d\ln\left(\Omega R/H\right)}$, for the entire apparent shear rate range of 0.5 to 100 s ^−1^. There are two distinct slopes, the first is valid for all gaps for torques less than 7 × 10^−4^ to 9 × 10^−4^ N-m and the second slope prevails above this range. Thus, for all gaps the slope $\frac{d\ln ℑ}{d\ln\left(\Omega R/H\right)}$ changes at the critical torque, ${ℑ}_{c}$ (Figure 5). For ℑ < ${ℑ}_{c}$, the slope $\frac{d\ln ℑ}{d\ln\left(\Omega R/H\right)}$ varies between 0.58 to 0.66 with a mean value of 0.62 (Figure 5). On the other hand, for $ℑ\geq {ℑ}_{c}$, the slope of the torque versus the apparent shear rate at the edge, i.e., $\frac{d\ln ℑ}{d\ln\left(\Omega R/H\right)}$ is 0.34 for *H* = 1 mm, $\frac{d\ln ℑ}{d\ln\left(\Omega R/H\right)}$ is 0.38 for *H* = 0.75 mm, and $\frac{d\ln ℑ}{d\ln\left(\Omega R/H\right)}$ is 0.44 for *H* = 0.5 mm. Thus, overall, there is a change in slope of the torque data at the critical torque from 0.62 ± 0.04 to 0.39 ± 0.05. Let us now see what this change in the slope of the torque versus the apparent shear rate represents.

The torque, ℑ, that is necessary to rotate the upper disk at a given apparent shear rate at the edge, ${\dot{\gamma }}_{aR}=\Omega R/H$, is given by:

(8) $$ℑ=2\pi \int_{0}^{R}\left(-\tau_{z\theta }\left(r\right)\right)r^{2}dr$$

Upon a change of variable of integration to:

(9) $$\frac{{\dot{\gamma }}_{aR}{}^{3}\;ℑ}{2\pi R^{3}}=\int_{0}^{{\dot{\gamma }}_{aR}}\left(-\tau_{z\theta }\left(r\right)\right){\dot{\gamma }}_{ar}{}^{2}d{\dot{\gamma }}_{ar}$$

and differentiation with respect to the apparent shear rate at the edge, ${\dot{\gamma }}_{aR}=\Omega R/H$, and utilizing the Leibniz rule of integration, one obtains the following relationship between the torque, versus the shear stress at the edge, *R* [101,102]:

(10) $$-\tau_{z\theta }\left(R\right)=\frac{ℑ}{2\pi R^{3}}\left(3+\frac{d\ln ℑ}{d\ln\left(\Omega R/H\right)}\right)$$

For the apparent slip mechanism and using a parallel-plate analogy, the shear stress $\tau_{z\theta }\left(r\right)$ at any radial position, *r*, in steady torsional flow can be determined (for the case of the top surface moving) using [27]:

(11) $${\left[\frac{-\left(\tau_{z\theta }\left(r\right)+\tau_{0}\right)}{m}\right]}^{1/n}\left(1-\frac{2\delta }{H}\right)+\frac{2\delta }{H}{\left(\frac{-\tau_{z\theta }\left(r\right)}{m_{b}}\right)}^{1/n_{b}}=\frac{\Omega r}{H}\;\mathrm{for}-\tau_{z\theta }\left(r\right)>\tau_{0}$$

(12) $$\frac{2\delta }{H}{\left(\frac{-\tau_{z\theta }\left(r\right)}{m_{b}}\right)}^{1/n_{b}}=\frac{\Omega r}{H}\;\mathrm{for}-\tau_{z\theta }\left(r\right)\leq \tau_{0}$$

Equations (11) and (12) indicate that the relationship between the shear stress, $\left|\tau_{z\theta }\left(r\right)\right|$ and the apparent shear rate expected for the pure plug flow, i.e., for $\left|\tau_{z\theta }\left(r\right)\right|\leq \tau_{0}$, would be different than the one that prevails under shear stresses for which $\left|\tau_{z\theta }\left(r\right)\right|>\tau_{0}$. How would this manifest itself for the torque, ℑ versus the apparent shear rate at the edge, ΩR/H, behavior and how different would the slope $\frac{d\ln ℑ}{d\ln\left(\Omega R/H\right)}$ be for the deformation region, i.e., $\left|\tau_{z\theta }\left(r\right)\right|>\tau_{0}$ in comparison to the plug flow region, i.e., $\left|\tau_{z\theta }\left(r\right)\right|\leq \tau_{0}$?

For the apparent wall slip mechanism the torque values for pure plug flow, i.e., $\left|\tau_{z\theta }\left(R\right)\right|\leq \tau_{0}$, can be determined as a function of the apparent shear rate at the edge of the disks, ${\dot{\gamma }}_{aR}=\Omega R/H$, as the following for a binder with a power-law type shear viscosity represented by the consistency index, *m_b_* and a power-law index, *n_b_* (for constant apparent slip layer thickness, *δ*):

(13) $$ℑ(r_{0}>R)=\frac{2\pi \;m_{b}R^{3}}{\left(3+n_{b}\right)}{\left(\frac{\Omega R}{2\delta }\right)}^{n_{b}}=\frac{2\pi \;m_{b}R^{3}H^{n_{b}}}{(3+n_{b})\;{\left(2\delta \right)}^{n_{b}}}{\left({\dot{\gamma }}_{aR}\right)}^{n_{b}}$$

Thus, for a non-Newtonian binder that constitutes the apparent slip layer with constant *δ*, and with shear viscosity represented by a power-law equation, the slope $\frac{d\ln ℑ}{d\ln\left(\Omega R/H\right)}$ would be equal to the power law index of the binder, *n_b_*. On the other hand, for a Newtonian binder with viscosity, $\mu_{b}$:

(14) $$ℑ(r_{0}>R)=\frac{\pi \;\mu_{b}R^{4}\Omega }{4\delta }=\frac{\pi \;\mu_{b}R^{3}H}{4\delta }{\dot{\gamma }}_{aR}\;\mathrm{and}\;\frac{d\ln ℑ}{d\ln\left(\Omega R/H\right)}=1.$$

It should be noted that the power law index of the binder, *n_b_* can also be determined from the wall slip analysis, i.e., from the relationship between wall slip velocity and shear stress (Section 2.4). In Section 2.4, it will be shown that the apparent slip layer formation can also be affected by the penetration of the macromolecules that are dangling from the surfaces of the crosslinked and swollen soft particles of PAA.

What happens if the shear stress, $\left|\tau_{z\theta }\left(r\right)\right|$ exceeds the yield stress at a radial position $r_{0}$, where $r_{0}$ is the radial location at which $\left|\tau_{z\theta }\left(r_{0}\right)\right|=\tau_{0}$ during steady torsional flow? Part of the viscoplastic fluid in between the two parallel disks would be undergoing plug flow (solid body motion), i.e., for $r\leq r_{0}$, and part of the fluid would be undergoing deformation for $r>r_{0}$, with a transition at $r=r_{0}$, i.e., $\left|\tau_{z\theta }\left(r_{0}\right)\right|=\tau_{0}$. For cases where there is plug flow and deformation at a constant rate occurring simultaneously, the torque, ℑ, can be determined as [99]:

(15) $$ℑ=2\pi \int_{0}^{R}\left(-\tau_{z\theta }\left(r\right)\right)r^{2}dr=2\pi \left[\int_{0}^{r_{0}}\left(-\tau_{z\theta }\left(r\right)\right)r^{2}dr+\int_{r_{0}}^{R}\left(-\tau_{z\theta }\left(r\right)\right)r^{2}dr\right]$$

The first term on the right is the contribution of the plug flow zone to the torque and is equal to:

(16) $$2\pi \int_{0}^{r_{0}}\left(-\tau_{z\theta }\left(r\right)\right)r^{2}dr=\frac{2\pi \;m_{b}r_{0}{}^{3+n_{b}}}{\left(3+n_{b}\right)}{\left(\frac{\Omega }{2\delta }\right)}^{n_{b}}$$

The torque for the deformation region has the contributions of the apparent slip as well as the bulk deformation of the hydrogel. However, as the shear stress increases and becomes significantly greater than the yield stress the contribution of wall slip diminishes. This will be shown in conjunction with the results and discussion available in Section 2.5. When the effect of the apparent slip diminishes with increasing shear stress, the slope becomes equal to the power law index, *n*, of the hydrogel, as shown below:

(17) $$\int_{r_{0}}^{R}\left(-\tau_{z\theta }\left(r\right)\right)r^{2}dr=\frac{\tau_{0}}{3}\left(R^{3}-r_{0}{}^{3}\right)+\frac{m}{\left(3+n\right)}{\left(\frac{\Omega }{H}\right)}^{n}\left(R^{3+n}-r_{0}{}^{3+n}\right)$$

so that the torque, ℑ, for the condition of negligible slip contribution in the deformation region, i.e., for shear stress significantly greater than yield stress, can be obtained as [99]:

(18) $$ℑ=2\pi \int_{0}^{R}\left(-\tau_{z\theta }\left(r\right)\right)r^{2}dr=2\pi \left[\frac{m_{b}r_{0}{}^{3+n_{b}}}{\left(3+n_{b}\right)}{\left(\frac{\Omega }{2\delta }\right)}^{n_{b}}+\frac{\tau_{0}}{3}\left(R^{3}-r_{0}{}^{3}\right)+\frac{m}{\left(3+n\right)}{\left(\frac{\Omega }{H}\right)}^{n}\left(R^{3+n}-r_{0}{}^{3+n}\right)\right]$$

The third term on the right side of Equation (18) dominates for *R* >> *r*_0_ so that $\frac{d\ln ℑ}{d\ln\left(\Omega R/H\right)}\approx n$ (Equations (1)–(4)). As shown in Section 2.4 and Section 2.6, the wall slip analysis followed by the determination of the parameters of the Herschel–Bulkley Equation for the hydrogel accurately provides the value of the shear rate sensitivity index of the hydrogel, *n*, so that it can be compared with the approximate value of the *n* value determined from the torque versus the rotational speed data discussed here.

Thus, in the steady torsional flow of the hydrogel subject to apparent wall slip, a slope change in the torque versus the rotational speed from the power law index of the binder, *n_b_*, to a value approaching the shear rate sensitivity index, *n*, of the Herschel–Bulkley fluid is expected. Typically, *n* < *n_b_*, considering that when a binder is mixed with soft (as in the hydrogel of this study) or rigid particles the resulting suspension is generally pseudoplastic in nature, i.e., *n <* 1. There are exceptions to this for dilatant suspensions for which *n* > 1. Such dilatant suspensions typically incorporate low-aspect particles with a narrow size range [30,31]. Regardless of the nature of the rheological behavior of the viscoplastic fluid versus the rheological behavior of the binder the change in slope reflects the transition from pure plug flow to a flow with both plug flow for *r* ≤ *r*_0_ and deformation flow for *r*_0_ < *r* ≤ *R*.

Therefore, the change in the slope, $\frac{d\ln ℑ}{d\ln\left(\Omega R/H\right)}$, is expected to occur when the shear stress at the edge becomes equal to the yield stress, i.e., $\left|\tau_{z\theta }\left(R\right)\right|=\tau_{0}$. Thus, this step change in the slope $\frac{d\ln ℑ}{d\ln\left(\Omega R/H\right)}$ serves as the basis for the determination of the yield stress, $\tau_{0}$, value of a viscoplastic fluid using steady torsional flow [99]. Overall, it is sufficient to collect torque, ℑ, versus rotational speed, Ω, data at a single gap, *H*, for the determination of the yield stress.

**Figure 6:**
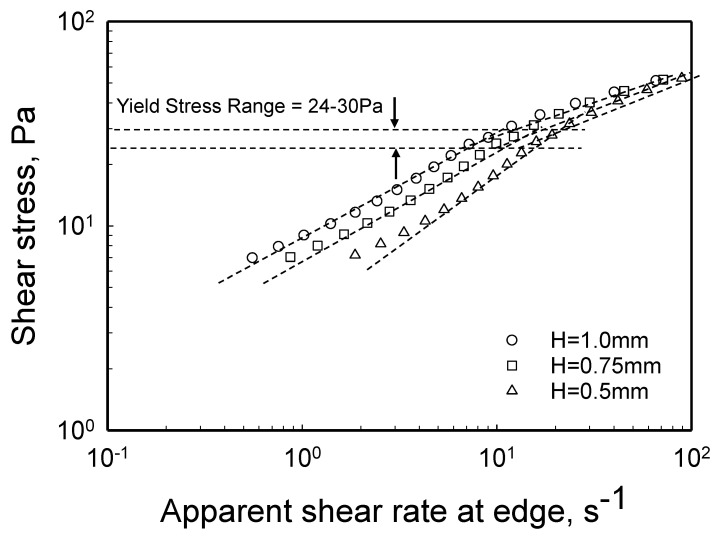
Shear stress at the edge, $\left|\tau_{z\theta }\left(R\right)\right|$, versus the apparent shear rate at the edge, ${\dot{\gamma }}_{aR}=\Omega R/H$, for three gaps, *H* = 1, 0.75 and 0.5 mm the yield stress range is indicated and corresponds to the critical torque range.

For the Carbopol^®^ hydrogel at 0.12% by weight, what is the critical shear stress range at the edge that corresponds to the critical torque range of 0.0007 ≤ ${ℑ}_{c}$ ≤ 0.0009 N-m? Applying Equation (10) for the critical condition, i.e., ${\left|\tau_{z\theta }\left(R\right)\right|}_{c}=\frac{{ℑ}_{c}}{2\pi R^{3}}\left(3+\frac{d\ln ℑ}{d\ln\left(\Omega R/H\right)}\right)$ using the mean value of $\frac{d\ln ℑ}{d\ln\left(\Omega R/H\right)}$ = 0.62 for $ℑ<{ℑ}_{c}$ (Figure 5) the critical shear stress range at the edge, ${\left|\tau_{z\theta }\left(R\right)\right|}_{c}$ is determined to be 24–30 Pa (Figure 6).

Thus, the yield stress, $\tau_{0}$, of the hydrogel is about 27 Pa, which is exactly what was determined as the yield stress of this Carbopol^®^ hydrogel from previous investigations using Couette flow [84] and vane-in-cup flow [92]. The new methodology that is applied here for the determination of the yield stress of a viscoplastic fluid using steady torsional flow was also tested earlier for a concentrated suspension of rigid particles mixed with a poly(dimethyl siloxane) binder [98]. In that investigation, the determined yield stress value using the torque versus apparent shear rate data from steady torsional flow was again found to be similar to the yield stress values of the concentrated suspension obtained using wall slip analysis, as well as using a straight-line marker method [40].

### 2.2. Apparent Slip Analysis

It was indicated earlier that for the conditions of the apparent slip layer thickness, *δ*, or the shear viscosity behavior of the fluid comprising the apparent slip layer thickness (for a power-law fluid consistency index, *m_b_*, and power-law index, *n_b_*) remaining the same over the rotational speed, Ω, range imposed during plug flow of the hydrogel, Equation (13) would be valid for the torque. This highlights that the torque would remain independent of the gap, *H*, used in the steady torsional flow, regardless of whether the binder fluid is Newtonian or non-Newtonian (note that $H{\dot{\gamma }}_{aR}=\Omega R$).

**Figure 7:**
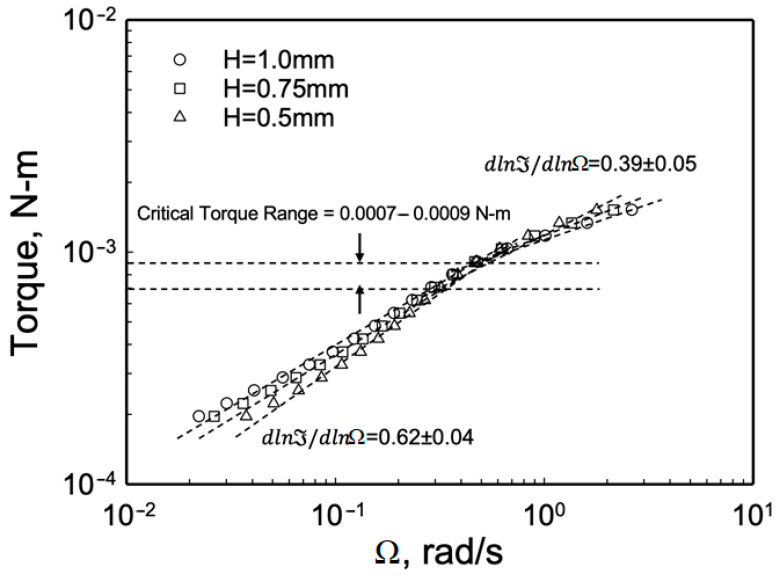
Steady torque, ℑ, versus the rotational speed, Ω, at the edge for three gaps.

However, as shown in Figure 7, there is dependence of the torque on the gap in the plug flow region, indicating that, either the slip layer thickness is changing, or that the rheological behavior of the fluid constituting the apparent slip layer is changing as the flow conditions are altered. Let us analyze the slip behavior in plug flow further.

In general, the wall slip velocity versus the shear stress behavior of complex fluids, including viscoplastic fluids, can be analyzed via systematic changes in the surface to volume ratio of the viscometer, i.e., by changing the gap, *H* [30,114,115] akin to the method suggested by Mooney for flow-through circular tubes [100]:

(19) $$\frac{\Omega r}{H}=\frac{U_{s}\left(r,0\right)}{H}-\frac{U_{s}\left(r,H\right)}{H}+\frac{dV_{\theta }}{dz}\left(r\right)$$

(20) $$\begin{array}{l} \frac{\Omega R}{H}=\frac{U_{s}\left(R,0\right)}{H}-\frac{U_{s}\left(R,H\right)}{H}+\frac{dV_{\theta }}{dz}\left(R\right) \end{array}$$

where $\frac{\Omega r}{H}$ is the apparent shear rate, ${\dot{\gamma }}_{ar}$, at the radial position, *r*, and $\frac{dV_{\theta }}{dz}\left(R\right)$ is the true shear rate, $\dot{\gamma }(R)$, imposed on the fluid at *r* = *R*, i.e., corresponding to the shear stress at the edge, $\tau_{z\theta }\left(R\right)$.

The slopes of the apparent shear rate with respect to 1/*H* at constant shear stresses provide the absolute values of the wall slip velocity at the given shear stresses so that one can obtain the slip velocity versus the shear stress behavior.

(21) ∂(ΩRH)∂(1H)|τzθ=2Us(τzθ(R))

Equation (21) suggests that if plots of apparent shear rate versus reciprocal gap are drawn at constant shear stress at the edge, the slopes would be equal to $2U_{s}\left(\tau_{z\theta }\left(R\right)\right)$, and extrapolated intercepts would be equal to the true shear rate at the edge. Yilmazer and Kalyon [30] have used more than two gaps and thus utilized Equation (20), whereas Yoshimura and Prud’homme have used only two gaps in their analysis [115], so that:

(22) $$U_{s}\left(\tau_{z\theta }\left(R\right)\right)=\pm R\frac{\left[\frac{\Omega_{1}\left(\tau_{z\theta }\left(R\right)\right)}{H_{1}}-\frac{\Omega_{2}\left(\tau_{z\theta }\left(R\right)\right)}{H_{2}}\right]}{2\left(\frac{1}{H_{1}}-\frac{1}{H_{2}}\right)}$$

where $\Omega_{1}\left(\tau_{z\theta }\left(R\right)\right)$ and $\Omega_{2}\left(\tau_{z\theta }\left(R\right)\right)$ are the rotational speeds for the two gaps, *H*_1_ and *H*_2_, at the same shear stress, $\tau_{z\theta }\left(R\right)$.

### 2.3. Plug Flow

**Figure 8:**
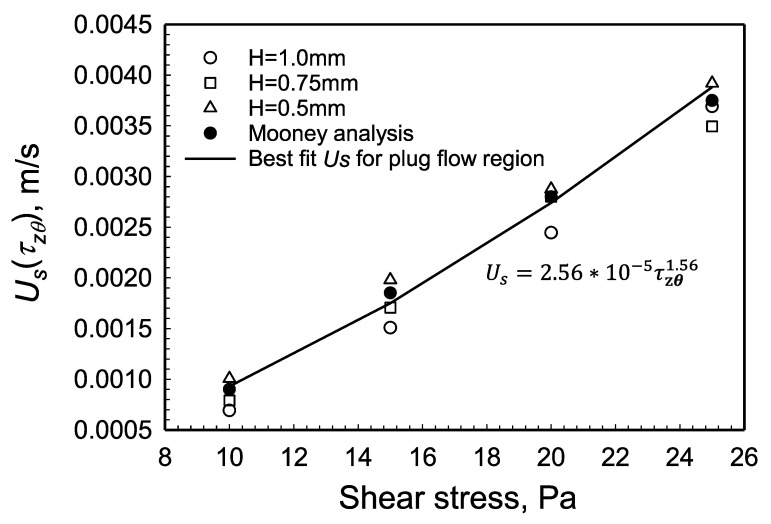
Wall slip velocity, $U_{s}$, versus shear stress at the edge, $\left|\tau_{z\theta }\left(R\right)\right|$, from the data collected at three gaps in the plug flow region.

Starting with Figure 6, the application of the analysis contained in Equation (20), i.e., for each gap, *H*, the apparent shear rate versus 1/*H* data were used at various shear stress values to determine the slopes which are equal to $2U_{s}$ to determine the relationship between slip velocity and shear stress at the edge. Figure 8 shows the slip velocity versus the shear stress behavior of the hydrogel determined in the plug flow region, i.e., $\left|\tau_{z\theta }\left(r\right)\right|\leq \tau_{0}$. The y-intercept in Equation (20) represents the true shear rate of the hydrogel. For plug flow, the y-intercept should be zero for data collected at all three gaps, indicating that plug flow prevails and the true shear rate is equal to zero. This expected behavior is indeed observed. As would be expected from the data shown in Figure 8, the slip velocity values obtained at different gaps, although they are close to each other, suggest some degree of dependence of the slip velocity values to the conditions generated at the different gaps that were used.

Meeker et al. have analyzed the formation of the apparent slip layer for gels with soft particles [29,90,91]. For plug flow formation in steady torsional flow, Meeker et al. have determined, based on Reynolds lubrication equation, that the apparent slip layer, *δ*, can be given as:

(23) $$\delta ={\left(\frac{\mu_{w}U_{s}R_{p}}{G_{p}}\right)}^{1/2}$$

where the Carbopol^®^ microgel with a Newtonian binder (water), with shear viscosity *μ_w_* consists of closely packed swollen soft particles with modulus of elasticity of *G_p_* and radius, *R_p_* [90,91]. $\tau_{z\theta }$ can be given as:

(24) $$\tau_{z\theta }=\frac{\mu_{w}U_{s}}{\delta }=\frac{\mu_{w}U_{s}}{{\left(\frac{\mu_{w}U_{s}R_{p}}{G_{p}}\right)}^{1/2}}={\left(\frac{\mu_{w}U_{s}G_{p}}{R_{p}}\right)}^{1/2}$$

and hence:

(25) $$\frac{\delta }{R_{p}}=\left(\frac{1}{G_{p}}\right)\tau_{z\theta }$$

Aktas et al. [29] have shown that for Carbopol^®^ hydrogels the ratio of *R_p_* over *G_p_* is a constant for the plug flow region, i.e., the apparent slip layer thickness varies linearly with the shear stress. A corollary of this finding is that the apparent slip velocity $U_{s}$ would vary with the square of the shear stress, i.e., $U_{s}=\tau_{z\theta }{}^{2}$ in the plug flow region. As shown in Figure 8 the exponent is in the range of 1.50 to 1.65, depending on the gap and the method used, and is thus smaller than 2, indicating that there is another mechanism at play.

### 2.4. Different Mechanisms of Apparent Slip for Plug Flow versus Deformation Region

**Figure 9:**
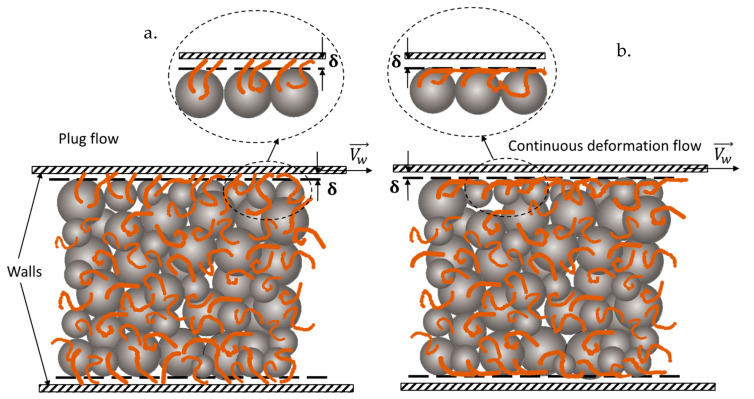
Hypothetical explanation of the differences in the shear viscosity of the fluid at the apparent slip layer, i.e., (**a**) penetration of the PAA chains into the slip layer for the plug flow region and (**b**) apparent slip layer free of particles and PAA chains for the continuous deformation region.

As indicated earlier in Equation (4) the relationship between the slip velocity, $U_{s}$, and the shear stress, $\tau_{z\theta }\left(R\right)$, for steady torsional flow is equal to $U_{s}\left(R\right)=\pm \beta {\left(-\tau_{z\theta }\left(R\right)\right)}^{s_{b}}$ [27], with ± necessary to accommodate the changing sign of the slip velocity at the stationary and moving walls, *β* is the slip coefficient and the reciprocal power law index, sb=1nb of the fluid that constitutes the apparent slip layer. For the gap dependency to be present in the above analysis, either the apparent slip layer thickness, *δ*, or the shear viscosity of the fluid constituting the apparent slip layer should change under different flow conditions, although all lead to plug flow of the hydrogel. The slope *s_b_* gives a hint as to what is happening. Considering that the binder of the gel is Newtonian water, and therefore *n_b_* = 1 and, hence, 1/*n_b_* = *s_b_* = 1. However, as seen in Figure 9, the value of the slope, *s_b_*, for plug flow is in the range of 1.5 to 1.65, and thus *n_b_* for the apparent slip layer in plug flow region is 0.60 to 0.67. It should be noted that the range of values of the power law index of the binder, *n_b_* = 0.6 to 0.67 agrees very well with the *n_b_* value obtained from the torque versus the rotational speed analysis, which had generated a *n_b_* value of 0.62 (Section 2.4).

The fact that the power law index of the binder *n_b_* is around 0.6 suggests that the fluid that constitutes the apparent slip layer for the plug flow region is non-Newtonian. What could impart a non-Newtonian character to the apparent slip layer, if the major constituent is water?

It is reasonable to assume that the soft, crosslinked, spherical PAA particles with dangling chains attached to their surfaces cannot come and pack efficiently at the wall as they can away from the wall. However, the free end of the PAA chains can penetrate into the apparent slip layer under the mild shear stress and shear rate conditions of plug flow, giving rise to a PAA solution at the apparent slip layer thickness. Thus, our hypothesis is that the dangling, poly(acrylic acid) (PAA) macromolecules of the Carbopol^®^ hydrogel, that are fixed to the crosslinked particles on one end, are able to rotate and orient freely on the other end. The chains would have some motion and orientation capabilities to penetrate into the apparent slip layer under plug flow conditions, as depicted schematically in Figure 9a.

**Figure 10:**
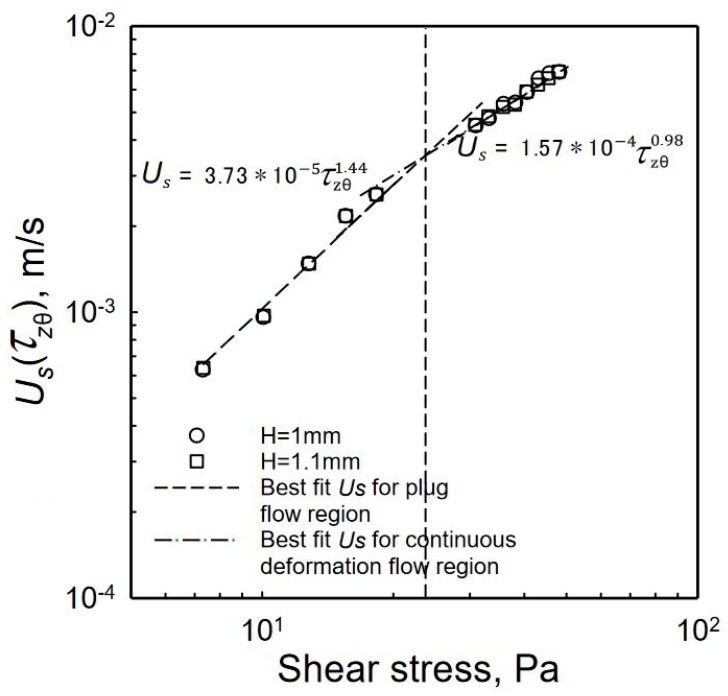
Wall slip velocity, $U_{s}$, versus shear stress at the edge, $\left|\tau_{z\theta }\left(R\right)\right|$, for two gaps from the data collected via PIV experiments [98]. The slope is equal to 3.73 × 10^−5^ m/(Pa^1.44^ s) for plug flow region and 1.573 × 10^−4^ m/(Pa^0.98^ s) for continuous deformation region.

On the other hand, for the continuous deformation region the slope *s_b_* ≈ 1 and hence the power law index of the fluid constituting the apparent slip layer, *n_b_ ≈* 1, characteristic of a Newtonian fluid (as would be expected here for the liquid phase, which is water, free of penetration of the dangling PAA chains into the apparent slip layer). This is shown in Figure 10, where it is indicated that for the data used involving the gap *H* = 1 and 1.1 mm, the relationship between the slip velocity and the shear stress is $U_{s}=1.57*10^{-4}\tau_{z\theta }{\left(R\right)}^{0.98}$.

It can be hypothesized that the higher shear stress and the shear rates found in the continuous deformation region of the steady torsional flow orient the macromolecules that are anchored to the soft particle surfaces, along the streamlines of the flow field (which are parallel to the wall velocity). This generates an apparent slip layer that is free of particles, as well as free from the presence of dangling PAA macromolecules (Figure 9b). Thus, only water constitutes the apparent slip layer for the continuous deformation region. Following up on this hypothesis, the apparent slip layer thickness, *δ*, for the continuous deformation region, comprised of water, can be determined using: $U_{s}=\beta *\left|\tau_{z\theta }\left(R\right)\right|=\frac{\delta }{\mu_{w}}*\left|\tau_{z\theta }\left(R\right)\right|$, i.e., $\delta =\beta *\mu_{w}=0.16\;\mu m$. This thickness determined for the continuous deformation region is a reasonable estimate of the apparent slip layer thickness, since the diameter of the soft crosslinked swollen PAA particles are estimated to be in the 2 to 3 μm range.

**Figure 11:**
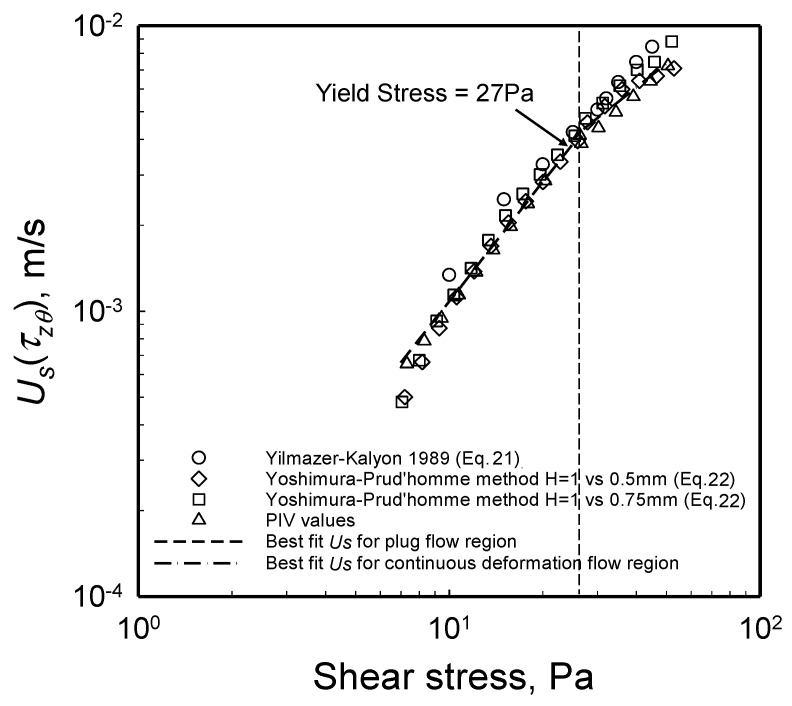
Slip velocity as a function of shear stress determined using Mooney procedures with best fits for the plug flow and deformation region that were reported in Figure 10.

It is interesting to compare the wall slip velocity values determined via the Mooney method with the wall slip velocity data obtained by Medina-Bañuelos et al., 2021 [98] employing PIV analysis. The comparisons are shown in Figure 11. The *β* value varies between 3.73 × 10^−5^ to 5.21 × 10^−5^ m/(Pa*^Sb^* s) and the exponent, *s_b_*, ranges from 1.34 to 1.44. The mean values of *β* and *s_b_* from these data are 4.50 × 10^−5^ and 1.39, respectively. Thus, the parameters of the wall slip velocity versus the shear stress relationship stay consistent for the different methods that are utilized.

### 2.5. Yield Stress from Wall Slip Analysis

**Figure 12:**
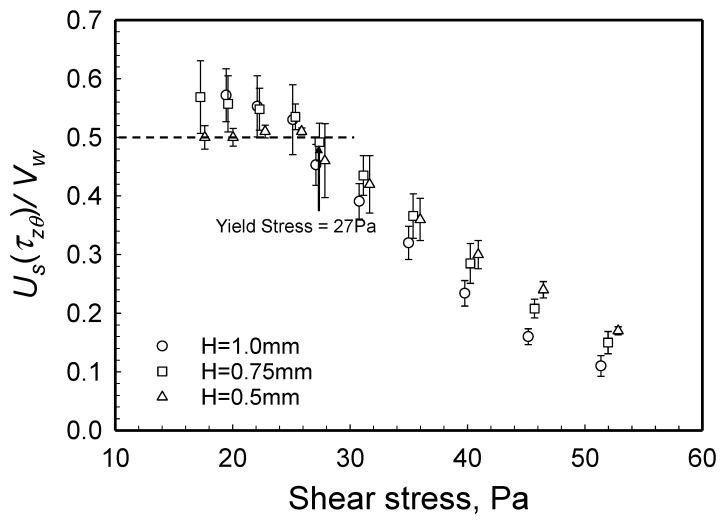
Wall slip velocity, $U_{s}$, over the plate velocity, *Vw* = ΩR,versus shear stress at the edge, $\left|\tau_{z\theta }\left(R\right)\right|$, from the data collected at three gaps. $\frac{U_{s}}{\Omega R}$ = 0.5 corresponds to plug flow.

To validate the yield stress value obtained with the torque versus the apparent shear rate data, one can also probe the relationship between the wall slip velocity and the velocity of the disk driving the steady torsional flow. The ratio of the wall slip velocity over the wall velocity at the edge, i.e., $\frac{U_{s}}{\Omega R}$ versus the shear stress at the edge, $\left|\tau_{z\theta }\left(R\right)\right|$, is shown in Figure 12. As indicated in Section 1.5.2, plug flow is indicated when the ratio $\frac{U_{s}}{\Omega R}$ = 0.5 [27]. The flow field changes from plug flow (for which $\frac{U_{s}}{\Omega R}$ = 0.5) to deformation flow (for which $\frac{U_{s}}{\Omega R}$ < 1) when the shear stress reaches the yield stress of the suspension [27]. As shown in Figure 12, this transition from plug flow to deformation flow occurs at the shear stress of 27 Pa, indicating that the yield stress of the hydrogel is 27 Pa. Thus, the yield stress value determined from wall slip analysis agrees with the yield stress determined from torque versus the apparent shear rate data. A third method, involving the velocity distributions obtained experimentally, as well as obtained upon computations with the parallel-plate analogy, was also applied and again generated a yield stress value close to 27 Pa. This third method will be discussed in Section 2.7. Overall, it should be noted that the use of the torque versus the rotational speed data is the simplest means to determine the yield stress value of a viscoplastic fluid.

### 2.6. Other Parameters of the Shear Viscosity of the Hydrogel Using the Herschel–Bulkley Equation

**Figure 13:**
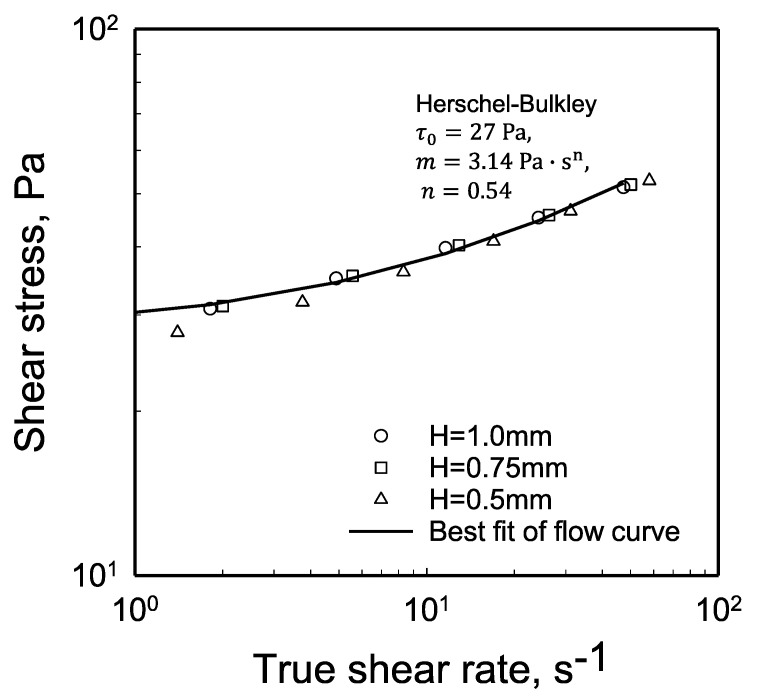
Shear stress at the edge, $\left|\tau_{z\theta }\left(R\right)\right|$, versus the true (slip corrected) shear rate at the edge, $\frac{dV_{\theta }}{dr}\left(R\right)=\dot{\gamma }(R)$, from the data collected at three gaps, *H*. The parameters of the Herschel–Bulkley Equation of the hydrogel are yield stress, $\tau_{0}$ = 27 Pa, *m* = 3.14 Pa-s^n^ and *n* = 0.54.

The shear stress at the edge versus the true shear rate at the edge for the three gaps are shown in Figure 13. Since the yield stress value of the hydrogel could be determined from the torque versus the apparent shear rate data directly, the other two parameters of the Herschel–Bulkley Equation could be readily obtained from the flow curves, i.e., the shear stress at the edge versus the true (slip corrected) shear rate at the edge. The best fit of the flow curve generated the other two parameters of the Herschel–Bulkley Equation as: *m* = 3.14 Pa-s^n^ and *n* = 0.54 (Figure 13). The shear viscosity of the hydrogel used here was characterized earlier using Couette and vane-in-cup flow, and its Herschel–Bulkley parameters were determined in these earlier investigations as $\tau_{0}$ = 27 Pa, *m* = 5.5 Pa-s^n^ and *n* = 0.43 [84,92]. The general agreement of the parameters obtained with different viscometric flows is indicative of the robustness of the methodologies used in determining the yield stress and the wall slip velocity versus shear stress behavior of the hydrogel from parallel-disk viscometry. As indicated earlier in Section 2.1, the shear rate sensitivity index of the hydrogel, *n*, was determined as 0.39 ± 0.05 from the torque versus the rotational speed analysis (see the continuous deformation region of Figure 5). The value of *n* determined from the analysis of the flow curve following the wall slip analysis (*n* = 0.54) should be considered more accurate than the value of *n* approximated from the torque versus rotational speed analysis (*n* = 0.39 ± 0.05). The *n* values determined with the two methods should approach each other as the applied shear stress becomes significantly greater than the yield stress of the hydrogel (as shown in Figure 12, the effect of wall slip diminishes as the shear stress is increased).

Table 1 shows the parameters of the wall slip velocity versus the shear stress for the plug flow and the continuous deformation regions, and the parameters of the shear viscosity of the hydrogel, employing the viscoplastic Herschel–Bulkley constitutive equation. These parameters were subjected to an additional test, involving the prediction of the velocity distributions and torques and their comparison with the experimental values, as discussed next.

### 2.7. Predictions of the Velocity Distributions and Comparisons with Experimental Distributions

**Figure 14:**
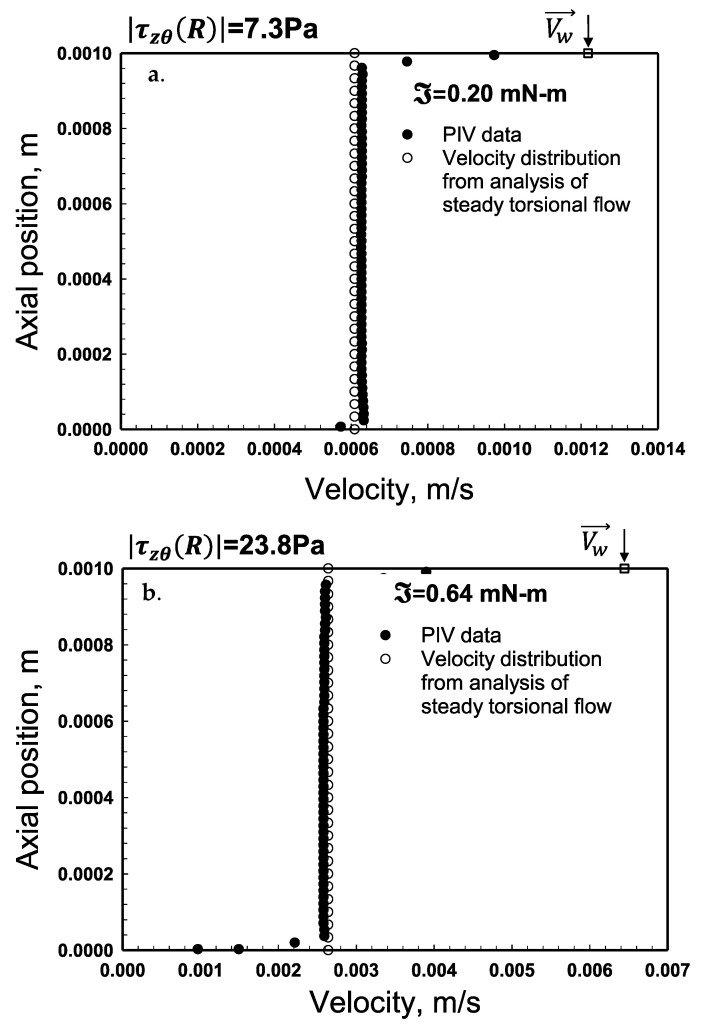
Velocity distributions in steady torsional flow using parallel disks for the torque, ℑ values of 0.20 mN-m (**a**) and 0.64 mN-m (**b**) for the gap, *H* = 1.0 mm. The PIV data are from Medina-Bañuelos et al., 2021. The shear stress values are 7.3 and 23.8 Pa.

**Figure 15:**
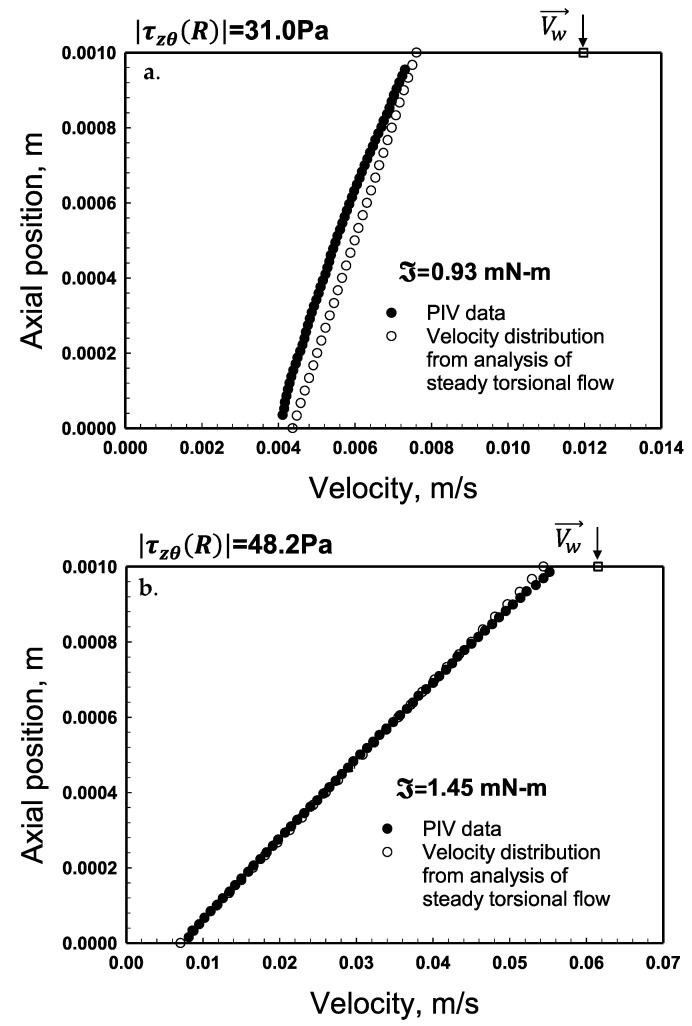
Velocity distributions in steady torsional flow using parallel disks for various ℑ values of (**a**) 0.93 and (**b**) 1.45 mN-m for the gap, *H* = 1.0 mm. The PIV data are from Medina-Bañuelos et al., 2021 [98].

**Figure A2:**
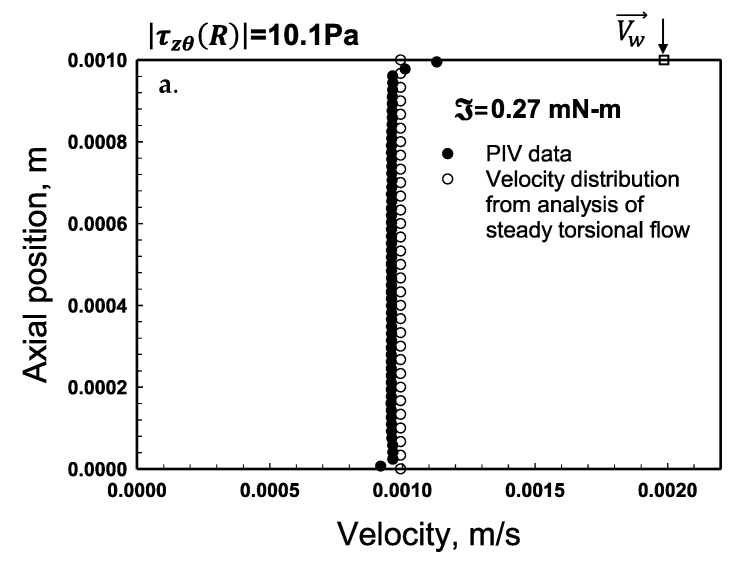

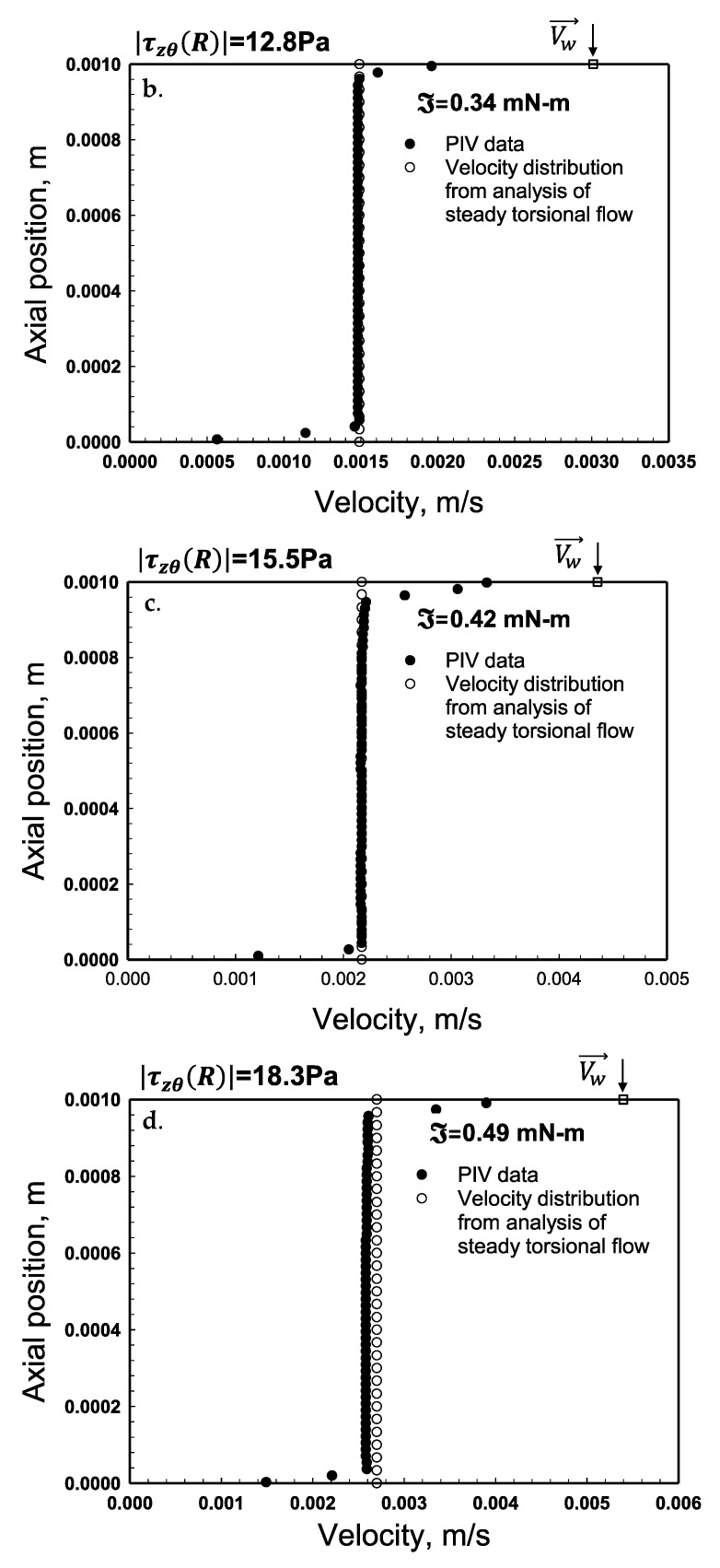
Plug flow with wall slip in steady torsional flow using parallel disks for the gap, *H* = 1.0 mm for the shear stress at the edge values of (**a**) 10.1 Pa, (**b**) 12.8 Pa, (**c**) 15.5 Pa, (**d**) 18.3 Pa. The PIV data are from Medina-Bañuelos et al., 2021. Filled symbols are from PIV data and unfilled are from calculations.

**Figure A3:**
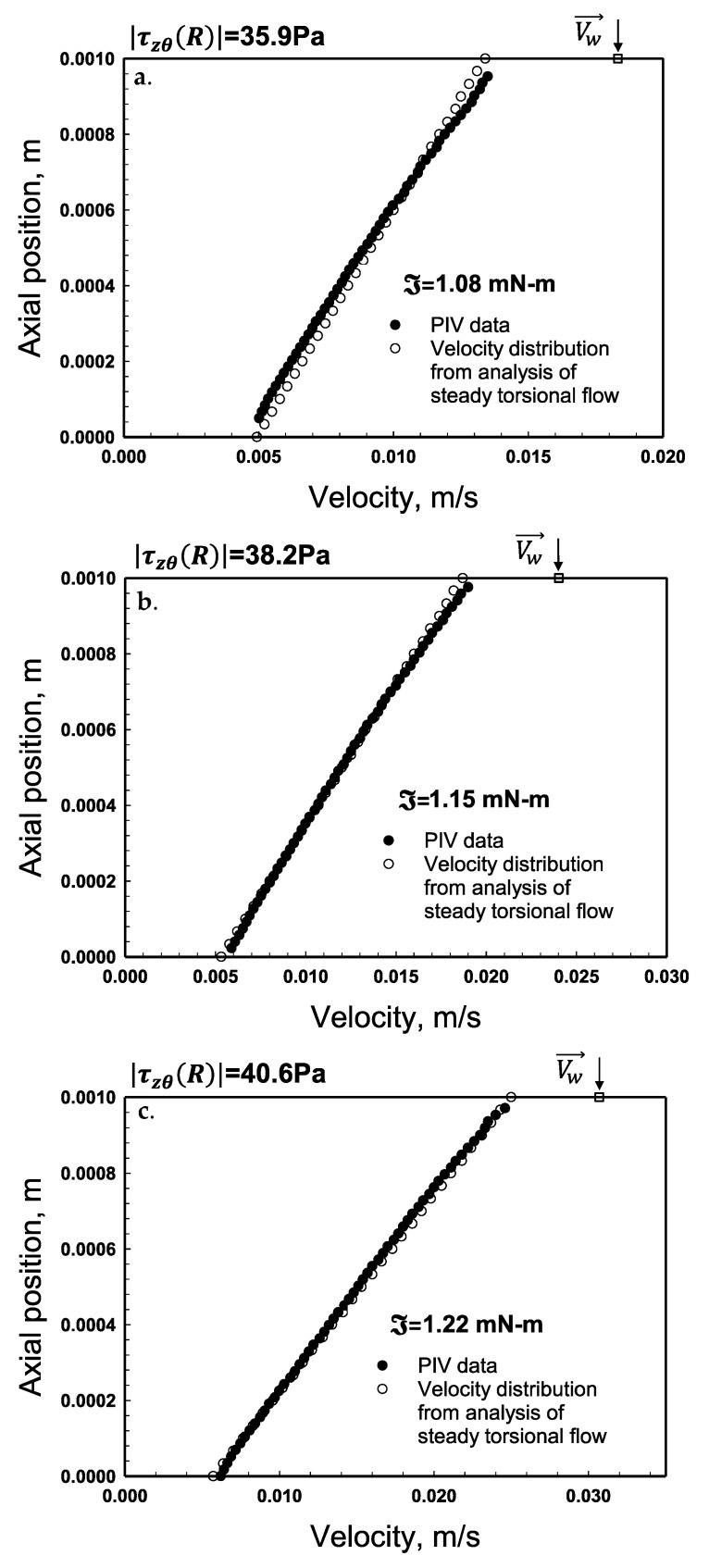

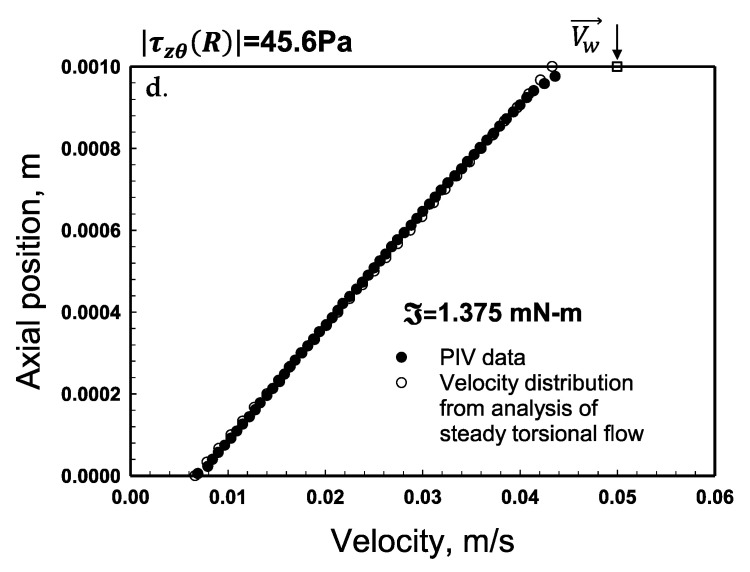
Velocity distributions with wall slip in steady torsional flow using parallel disks for the gap, *H* = 1.0 mm for the shear stress at the edge values of (**a**) 35.9 Pa, (**b**) 38.2 Pa, (**c**) 40.6 Pa, (**d**) 45.6 Pa. The PIV data are from Medina-Bañuelos et al., 2021. Filled symbols are from PIV data and unfilled are from calculations.

The velocity distributions that Pérez-González and co-workers, 2021, collected using the PIV method are shown in Figure 14 and Figure 15 and in Figure A2 and Figure A3 in Appendix B. The velocity distributions were obtained for *H* = 1.0 mm for each ℑ value. The experimental velocity distributions were compared with the predictions of the velocity distributions, relying on parallel-plate analysis (Equations (11) and (12)). Figure 14 shows the experimental and predicted velocity distributions for the torque, ℑ, values of 0.2 and 0.64 mN-m, whereby the corresponding shear stress values are 7.3 and 23.8 Pa. As expected, considering that the shear stress values are less than the yield stress of the hydrogel the tangential velocity values are constant in between the two plates, i.e., the flow of the hydrogel is plug flow (Figure 14 and Appendix B Figure A2). This is consistent with how viscoplastic fluids flow when the imposed shear stress is less than the yield stress of the fluid. The absolute values of the wall slip velocities, experimentally determined at the top and bottom walls, were similar to each other. As an example, the measured values of the slip velocities for the torque, ℑ, value of 0.2 mN-m were $U_{s}\left(R_{m},0\right)=6.28\times 10^{-4}m/s$ and $U_{s}\left(R_{m},H\right)=-6.22\times 10^{-4}m/s$ at the bottom and top walls, respectively. These slip velocities at the two walls indeed generate the following as required for plug flow:

(26) $$U_{s}\left(R_{m},0\right)=-U_{s}\left(R_{m},H\right)=\Omega R_{m}/2$$

On the other hand, the calculations and the experimental data for the velocity distributions corresponding to torques of 0.93 and 1.45 mN-m (shear stress values of 31 and 48.2 Pa) are shown in Figure 15. As expected, when the shear stress exceeds the yield stress there is constant steady deformation flow (Figure 15a,b). It is observed that for $\left|\tau_{z\theta }\left(r\right)\right|>\tau_{0}$, the tangential velocity, *V_θ_* (*r*) increases linearly with axial distance, *z* and the constant deformation rate, $\frac{dV_{\theta }}{dz}\left(r\right)$ increases with increasing rotational speed, Ω. Similar to the plug flow case, apparent wall slip plays a key role, and the absolute values of the wall slip velocities at the top and bottom walls are equal to each other.

Additional experimental data and predictions are presented in Appendix B (Figure A3). The excellent agreement between the experimental distributions and the numerical simulation results suggest that the parallel-plate analysis is satisfactory to represent the flow and deformation occurring in steady torsional flow and that the parameters of shear viscosity and wall slip are accurate.

Can the velocity distributions (Figure 14 and Figure 15) be used to bracket the yield stress value? The plug flow observed in Figure 14b occurs at a shear stress of 24 Pa, whereas the continuous deformation profile shown in Figure 15a occurs at a shear stress of 30 Pa. Therefore, the experimental velocity distributions suggest that the yield stress of the hydrogel is between 24 and 30 Pa, consistent with the yield stress obtained using the torque versus apparent shear rate data (Figure 5 and Figure 6) which had identified the same shear stress range for the yield stress.

### 2.8. Predictions of the Torques at Various Rotational Speeds and Comparisons with Experimental Torque Values

To test further the accuracy of the parameters of wall slip velocity versus the shear stress relationship and the shear viscosity material function of the hydrogel, the torques under different conditions were also solved by numerical integration. For this the parallel-plate approximation was again used, i.e., Equations (11) and (12), which were solved incrementally in the radial direction for each set of disk rotational speed, Ω, and gap, *H*, via numerical integration using the MATLAB code. This additional step of the prediction of the torques and their comparisons with the experimental torque values of Medina-Bañuelos et al., 2021, provides an additional assessment of the accuracy of the parameters of shear viscosity and wall slip. The first step in this procedure is the determination of the shear stress distribution as a function of the radial position, *r*.

**Figure 16:**
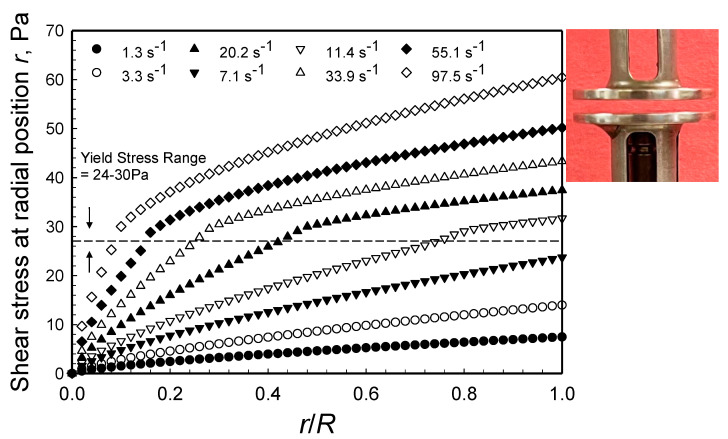
Radial distributions of apparent shear stress, $\left|\tau_{z\theta }\left(r\right)\right|$ in steady torsional flow between two disks for the range of apparent shear rates at edge, ${\dot{\gamma }}_{a\;R}=\Omega R/H$, between 1.3 to 97.5 s^−1^ (*H* = 1 mm).

The shear stress distributions, $\tau_{z\theta }\left(r\right)$, for various apparent shear rates as a function of the radial position, *r*, (at gap, *H* = 1 mm), are shown in Figure 16 for various apparent shear rates. The shear stress increases monotonically with increasing *r*, reaching a maximum at the edge of the disk. There is a significant difference in the shear stress distribution obtained under the plug flow conditions (apparent shear rates in the 1.3 to 7.1 s^−1^) and the deformation flow conditions (apparent shear rates in the 11.4 to 97.5 s^−1^). The yield stress range of 24–30 Pa clearly delineates the shear stress distributions into the expected two zones related to plug flow and the deformation flow conditions.

**Figure A4:**
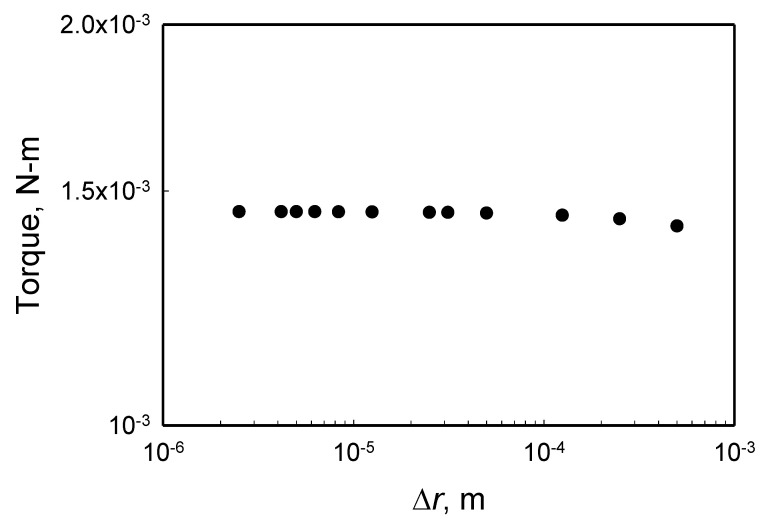
The effects of the radial distance increment Δ*r* on the torques calculated using numerical integration for the conditions of *H* = 0.001 m and Ω = 2 rad/s.

Upon calculation of the shear stress distribution, $\tau_{z\theta }\left(r\right)$, the torque, ℑ, at each apparent shear rate $ℑ(H,\;\Omega )=2\pi \int_{0}^{R}\left(-\tau_{z\theta }\left(r\right)\right)r^{2}dr$ was obtained via numerical integration, via $ℑ(r)=2\pi \left(-\tau_{z\theta }\left(r\right)\right)r^{2}\Delta r$. The typical Δ*r* values were around 0.0005 m for *R* = 0.025 m, i.e., Δ*r*/*R* was 0.02. The effect of the choice of Δ*r* was probed by systematically changing Δ*r* in the 0.0005 to 0.00005 m range. As shown in Appendix C (Figure A4), the torque results converge and Δ*r* is no longer a factor when Δ*r* is smaller than 0.0005 m.

**Figure 17:**
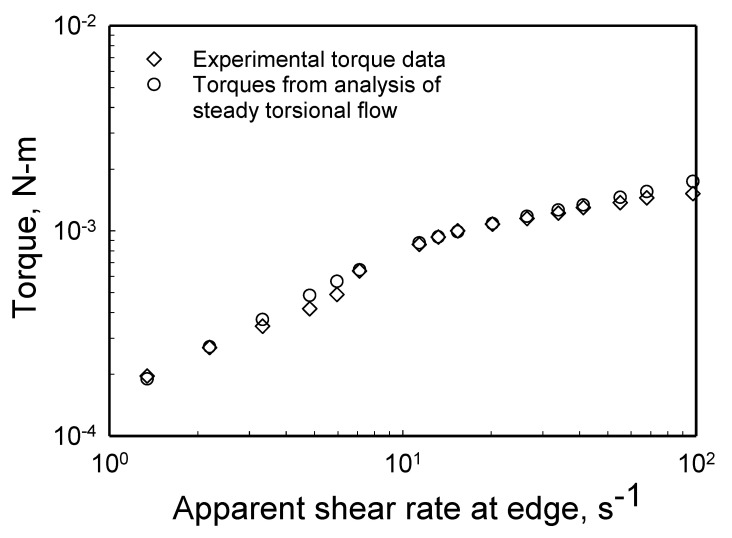
The comparisons of the converged torques obtained via numerical integration employing the characterized parameters of wall slip and shear viscosity with the experimental torque values.

The comparisons of the converged torques obtained via numerical integration, employing the characterized parameters of wall slip and shear viscosity (Table 1) with the experimental torque values, are shown in Figure 17. There is excellent agreement between the experimental torque values and those that were numerically determined (Figure 17). The excellent agreement is an additional testament that the parallel-plate approximation made under the condition of *H* << *R* (lubrication assumption) is acceptable and that the parameters of the shear viscosity and the slip velocity behavior are accurate.

## 3. Conclusions

The flow and deformation behavior of hydrogels is central to many of the applications that they are used for in myriad areas, as diverse as biomedical devices, hydraulic fracturing, foodstuffs, and personal care products. It is very important to be able to characterize, reproducibly and accurately, the rheological behavior of hydrogels using steady simple shear flows so that the flow and deformation behavior of the hydrogel can be readily understood and, if necessary, further tailored to the requirements of the application at hand. It is the viscoplasticity and the slip at the wall behavior of the hydrogels that render such characterization and tailoring difficult. Here, one of the simplest rheological characterization methods, parallel-disk viscometry, i.e., the steady torsional flow using parallel disks, is analyzed in detail. The analysis was carried out on a Carbopol^®^ hydrogel (0.12% by weight poly(acrylic acid)). A new method, involving the analysis of the torque versus the apparent shear rate data obtained from parallel-disk viscometry, is introduced. The analysis reveals that the hydrogel is viscoplastic with a yield stress in the range of 24–30 Pa (mean 27 Pa). This yield stress value is consistent with earlier investigations that relied on other types of viscometric flows including Couette flow in between two concentric cylinders and vane-in-cup flow. It is demonstrated that the method for the determination of the yield stress value of viscoplastic fluids in general, and the hydrogel in particular, via parallel-disk viscometry, is very simple to implement, and relies only on the collection of the torque versus the rotational speed data. It is shown that once the yield stress value is determined, the other parameters of viscoplastic constitutive equations, including the Herschel–Bulkley fluid, can be determined following analysis of the wall slip behavior of the hydrogel.

The analysis of the wall slip velocity versus the shear stress behavior of the hydrogel was carried out in conjunction with the apparent slip mechanism, i.e., the formation of a particle free binder-rich zone at the two walls of the viscometer. Such an apparent slip mechanism is widely encountered for concentrated suspensions and gels. It is determined that the mechanisms for the formation of the apparent slip layer thickness are different when the hydrogel is undergoing plug flow ($\left|\tau_{z\theta }\left(R\right)\right|\leq \tau_{0}$) or continuous deformation flow, i.e., $\left|\tau_{z\theta }\left(r\right)\right|>\tau_{0}$. The results indicate that the apparent slip layer consists of only water for the continuous deformation flow region. However, a complex behavior is observed for the plug flow region. PAA chains are attached firmly to the particles at one end and are free, “dangling” to rotate and orient at their free end. It is hypothesized that the dangling PAA chains could penetrate the apparent slip layer under the relatively low shear stresses of the plug flow region to render the fluid found at the apparent slip layer non-Newtonian. It is hypothesized that at the higher shear stresses of the continuous deformation region, the dangling chains would orient along the streamlines, and clear away from the apparent slip layer, thus leaving only water to constitute the slip layer.

Following determination of the yield stress of the hydrogel from the torque versus the apparent shear rate data, the application of systematic changes in the surface-to-volume ratio of the parallel-disk viscometer allows the determination of the wall slip velocity versus the shear stress relationship, followed by the determination of the consistency index, *m*, and shear rate sensitivity exponent (power law index), *n*, of the Herschel–Bulkley fluid. The parameters of the shear viscosity and the apparent wall slip thus obtained were tested by being used for the prediction of the velocity distributions and the torques obtained under different flow conditions (employing a simple parallel-plate approximation in conjunction with the lubrication assumption). The predicted velocity distributions and the torque values were compared with the experimental data of Pérez-González and co-workers [99]. The excellent agreement between the predicted and experimentally determined torque values and the velocity distributions are testaments to the reliability of the determined parameters and the suitability of parallel-plate flow approximation-based methods for the analysis of parallel disk viscometry flow.

## Figures and Tables

**Table 1:** The parameters of wall slip and shear viscosity.

Slip velocity versus shear stress	$U_{s}\left(R\right)=\pm \beta {\left(-\tau_{z\theta }\left(R\right)\right)}^{s_{b}}$
Plug flow region	Deformation flow region
*β* = 2.56 × 10^−5^ m/(Pa^Sb^ s), *s_b_* = 1.56	*β* = 1.57 × 10^−4^ m/(Pa^Sb^ s), *s_b_* = 0.98
Herschel-Bulkley Equation (Equation (4))	
$\tau_{0}$ = 27 Pa, *m* = 3.14 Pa-s^n^ and *n* = 0.54	

**Figure A1:**
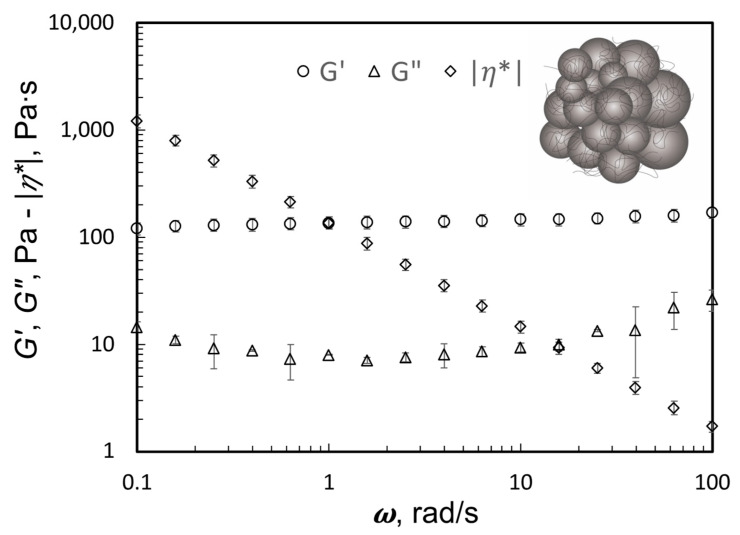
The dynamic properties (linear viscoelastic material functions of storage moduli, *G*′, loss moduli, *G*″, and the magnitude of complex viscosity, ∣η*∣) of the Carbopol^®^ hydrogel at a concentration of 0.12% by weight at 25 °C.

## Data Availability

Not applicable.

## Appendix A. Materials and Methods

### Appendix A.1. Materials

Carbopol^®^ hydrogels (referred to as “microgels” in some of our earlier publications) were prepared by dissolving different concentrations of Carbopol^®^ 940 in water. A concentration of 0.12% by weight of freshly procured Carbopol^®^ 940 in water was used. Comparisons of consecutive investigations revealed that freshness is essential for reproducibility of the rheology data. Carbopol^®^ was dissolved in tri-distilled water under continuous stirring [84]. Hollow glass particles (Potters Industries) of 10 μm in size and having a specific gravity of 1.1 ± 0.05 were added into the dispersion at a concentration of 0.03 wt.% to serve as flow tracers. The hydrogel samples prepared this way at Stevens were subjected to the characterization of their dynamic properties (as reported below), and those prepared at Instituto Politéchnico Nacional of Mexico were used by Medina-Bañuelos et al., 2021 to generate the experimentally obtained torques and velocity distributions. For the soft particles of the Carbopol^®^ hydrogel with particle radii *a* (see Figure 1 and Figure 2 for Carbopol^®^ particles), density, *ρ*, with binder viscosity, *μ_b_*, subject to shear rate $\dot{\gamma }$, with thermal energy, *kT*, the typical particle Reynolds number, *Re*, and the Peclet number, *Pe*, were determined to be Re(γ˙)=ρa2γ˙μb<<1, $Pe(\dot{\gamma })=\frac{6\pi \mu_{b}a^{3}\dot{\gamma }}{kT}>>1$, suggesting that the steady torsional flow of the Carbopol^®^ hydrogel does take place under the creeping flow regime and that the viscous forces dominate over the colloidal forces over the entire apparent shear rate, $\dot{\gamma }$, range of the experiments.

### Appendix A.2. Linear Viscoelastic Material Functions of the Carbopol^®^ Hydrogel (0.12%)

The storage, *G*′, and the loss modulus, *G*″, values of the Carbopol^®^ hydrogel were characterized at 25 °C (0.1–100 rad/s) employing an ARES rheometer from TA Instruments. The results are shown in Figure A1. The storage and loss moduli were found to be in the ranges of 120–170 and 14–52 Pa, respectively, as shown in Figure A1.

Thus, the storage moduli were about one order of magnitude greater than the loss modulus values, i.e., *G*′ >> *G*″. It was also determined that, consistent with earlier reports of the dynamic properties of the Carbopol^®^ hydrogel [84], the dynamic properties of the storage, *G*′, and the loss modulus, *G*″, values were only very weak functions of the frequency of the oscillatory deformation, *ω*. These two fingerprints of the linear viscoelastic properties, such as *G*′ >> *G*″ (tan*δ* = *G*″/*G*′ between 0.05 and 0.1) and *G*′ and *G*″ ≠ *f*(*ω*), reflect the typical behavior of gels and gel-like materials [16,17,45,108,116]. Such plateau behavior of *G*′ ≠ *f*(*ω*) and *G*″ ≠ *f*(*ω*), which spans a broad range of frequencies, suggests the formation of a reversible network structure typically characterized by a yield stress [16,17,45]. It should be noted that the interactions between the soft particles of crosslinked poly(acrylic acid) and the entanglements of the chains of poly(acrylic acid) that are dangling from the surfaces of the crosslinked particles generate the gel-like behavior and hence the yield stress of the hydrogel.

### Appendix A.3. Experimental Equipment and Procedures Used by Medina-Bañuelos and Co-Workers

The experiments of Medina-Bañuelos et al. were carried out using rheo-PIV measurements [98], the rotational rheometer was used in the parallel-disk mode with the sample sandwiched in between two parallel disks. All steady torsional flow measurements were performed at four different gap values, i.e., *H* = 0.5 and 0.75, 1.0, and 1.1 mm. The flow curves were obtained by controlling the torque (N-m), in a step-like ramp, and waiting for the attainment of a steady state at each step to record the angular velocity of the upper plate that would correspond to the imposed torque [98]. The PIV data for the steady torsional flow were collected in the *θ**z* plane, as close to the edge of the plates as possible, i.e., at *R_m_* = 2.35 mm, where *R_m_* is the measuring position of the velocity distributions, defined as the distance from the edge of the disks. Additional information can be obtained from [98].

## Appendix C. The Effect of the Choice of Δ*r*

Figure A4 shows the torque values, determined numerically using the parameters of wall slip velocity and the shear viscosity of the hydrogel (Table 1), in conjunction with the parallel plate approximation, as a function of Δ*r*. The predicted torque values become independent of Δ*r* when it is small enough. Torque values were obtained at a gap of 1 mm and rotational speed of 2 rad/s using our MATLAB code.
